# Neural and behavioral correlates of consciousness: the welfare consequences of fatal hypobaric and hypercapnic hypoxia in laboratory mice

**DOI:** 10.1038/s41684-026-01788-5

**Published:** 2026-08-21

**Authors:** Jasmine M. Clarkson, Dorothy E. F. McKeegan, Ross S. Muers, Matthew C. Leach, Julian Sparrey, Alfredo Gonzalez-Sulser, Jessica E. Martin

**Affiliations:** 1 School of Biodiversity, One Health and Veterinary Medicine, College of Medical Veterinary and Life Sciences, University of Glasgow, Glasgow, UK; 2 School of Natural and Environmental Sciences, Newcastle University, Newcastle Upon Tyne, UK; 3 Centre for Neuroscience, School of Psychology and Neuroscience, College of Medical Veterinary and Life Sciences, University of Glasgow, Glasgow, UK; 4 Scotland’s Rural College (SRUC), Edinburgh, UK; 5 Comparative Biology Centre, Newcastle University, Newcastle Upon Tyne, UK; 6 Livetec Systems Ltd, Wrest Park, Silsoe, UK; 7 Simons Initiative for the Developing Brain, Patrick Wild Centre and Centre for Discovery Brain Sciences, The University of Edinburgh, Edinburgh, UK; 8 The Royal (Dick) School of Veterinary Studies, The Roslin Institute, The University of Edinburgh, Edinburgh, UK

**Keywords:** Mouse, Consciousness, Ethics

## Abstract

Understanding an animal’s capacity to experience negative sensations, such as pain and distress, during impaired states of consciousness is critical in many contexts, particularly when animals are being euthanized. Mice remain the predominant species used for scientific research globally, where a prerequisite requires them to be humanely killed at the end of the scientific work. However, whether some of the existing practices and methodologies (for example, exposure to a rising concentration of carbon dioxide) provide these animals with a humane death has been questioned, thus requiring the development of novel, refined methodologies. Here we highlight the potential of hypobaric hypoxia to provide a humane alternative to hypercapnic hypoxia by combining behavioral and neurophysiological assessment (using electroencephalography) to characterize brain activity during the induction of unconsciousness. We found different patterns of brain activity between hypobaric and hypercapnic hypoxia, suggestive of key mechanistic differences in the transition toward unconsciousness. Unlike hypercapnic hypoxia, hypobaric hypoxia resulted in an early shift in peak power to lower delta frequency bands (<4 Hz), suggestive of early cognitive impairment and an anesthetic-like state before loss of posture. As such, mice are likely to have a reduced capacity to have negatively valenced sensory–affective experiences. Hypobaric hypoxia could provide the basis for major refinement to essential killing practices for laboratory mice worldwide.

## Abstract (web summary)

This study evaluates hypobaric hypoxia as a potential alternative to carbon dioxide euthanasia in mice. Data revealed earlier cognitive impairment, potentially reducing negative experiences and supporting a more humane killing method.

## Main

Understanding the capacity of animals to experience negatively valenced affective states, including pain, fear and anxiety, during impaired states of consciousness is relevant to a wide variety of situations, and is based on wide acceptance that many animals experience affective states and so are generally regarded as sentient (for example, Animal Welfare (Sentience) Act 2022^1^). The term ‘sensory–affective experiences’ is used here to reflect the integration of sensory input (that is, ‘sensation’) with immediate affective unpleasantness that characterizes many aversive states (that is, ‘affective states’) while avoiding assumptions that such experiences correspond directly to discrete, human-like emotional categories or higher-order emotional cognition^2,3^. This terminology is intended to acknowledge affective valence as central to welfare, without conflating it with more complex emotional constructs.

In humans, pain perception and other aversive experiences in those with disorders of consciousness (for example, individuals in a minimally conscious state), remain a matter of debate but are highly relevant for informing ethical obligations surrounding therapeutic action^4^. Determining states of consciousness in nonhuman animals is also vital for assessing their welfare during euthanasia, killing and slaughter, especially because, in most cases, death is not instantaneous. Comprehensive welfare assessment involves the triangulation of several approaches including measuring behavioral and physiological markers and comparing novel and existing protocols. Such studies are the basis of evidence-based recommendations to ensure the provision of a humane death for managed animals.

Assessing brain function and relating this to the likely degree of consciousness has been attempted using a variety of methods including measuring neural activity with an electroencephalogram (EEG). Data acquired from EEG provide an insight into global electrical brain activity and represent the combined output of synchronously firing populations of neurons^5^. EEG monitoring has been utilized in both human and veterinary neurology to ensure optimal levels of unconsciousness are maintained throughout surgical procedures. Normally this involves using an easily interpretable proxy (for example, bispectral index) to determine the depth of anesthesia^6–9^. During the transition from a conscious to an unconscious state (for example, during sleep or anesthesia), EEG activity changes from a signal dominated by low-amplitude, high-frequency activity to one with a relatively low-frequency, high-amplitude signal (commonly referred to as slow-wave activity)^7,9–11^. These changes have been repeatedly observed and validated in several species, including laboratory mice^12–14^.

Mice remain the predominant mammalian species used for biomedical research, with an estimated 192 million mice used globally each year^15^. An important prerequisite is that the majority must be killed upon completion of the scientific work or at the end of their breeding life^16,17^. The provision of the most humane death possible for these animals is not only a legal requirement, but also fulfills widely agreed moral obligations, vital for continued public acceptance and support for the use of animals for scientific purpose^18–20^.

Exposure to carbon dioxide (CO_2_) gas (inducing hypercapnic hypoxia) remains one of the most used methods to kill laboratory rodents^21^ despite substantial concerns for animal welfare due to its ability to induce pain^22,23^, fear^24,25^, anxiety^26,27^ and breathlessness^3,28,29^. A substantial body of evidence demonstrates the presence of negatively valenced sensory–affective experiences in animals during CO_2_ exposure, which has been extensively reviewed elsewhere^29–33^. Furthermore, our recent survey highlighted that CO_2_ killing protocols are widely variable in operational use (with regard to the fill method and flow rate) and often do not follow published guidance^21,29,32,34^. Most respondents reported filling the chamber from the bottom, as well as exceeding the recommended flow rate of 20% of the chamber volume per minute^32,34,35^ (for the UK and Europe)^21^. This is contrary to best practice, which recommends this flow rate and delivering the gas to the top of the chamber to avoid exposure to high concentrations of CO_2_ in conscious mice^32,34,35^. Existing legislation in the UK^16^ and Europe^36^ only defines “exposure to carbon dioxide gas in a rising concentration”, probably creating ambiguity in appropriate operational use. Exposure to CO_2_ induces a combination of both hypercapnic and hypoxic effects, as the delivery of CO_2_ into a sealed environment (that is, induction box/chamber) results in the displacement of oxygen. In laboratory rodents, CO_2_ concentrations exceeding 25% are considered noxious and as such, are widely used as pain stimuli in research^37–39^. At concentrations of 40% and above, CO_2_ reacts with water (H_2_O) in the mucous membranes to form carbonic acid (H_2_CO_3_), resulting in mucosal acidification that activates nociceptors^37–39^. The guidelines for the USA share recommendations of gradual exposure^40^. However, following their recent review, the recommended flow rate was increased to 30–70% for rodents in 2020^40,41^, citing conflicting and limited evidence that slower flow rates are less humane^40,42,43^. In addition, a conceivable serious negative impact on the animal during exposure to CO_2_ is its potential to induce concerning sensory–affective experiences such as breathlessness and ‘air hunger’^3^ (described as an urgent urge to breathe and the most unpleasant form of breathlessness^28^), even in the absence of higher-order emotional cognition.

An alternative approach that has been investigated in recent years is hypobaric hypoxia by the process of gradual decompression^11,44–46^. Hypobaric hypoxia describes a process of progressive reduction in air pressure, equivalent to ascending to a high altitude^47^. In gradual application, the resultant hypoxia is insidious and widely documented to have deleterious effects on cognitive function in humans, resulting in death if sustained^47^. Widely studied within aviation medicine, affected individuals become increasingly unaware that they are dangerously hypoxic and are incapable of acting accordingly^47–49^. Whether nonhuman mammals experience hypobaric hypoxia in the same way as humans before loss of consciousness is unknown, but evidence from birds suggests similar minimal adverse effects^11,50,51^.

Our previous work^44^ focused on the assessment of spontaneous behavior of laboratory mice while using pharmacological interventions to help infer their likely motivations (for example, expressed due to pain and/or anxiety) while undergoing gradual decompression or exposure to CO_2_ at the UK/European Union (EU)-recommended flow rate^34^. Despite elongated latencies to loss of posture (LOP) and death, mice exposed to hypobaric hypoxia still performed species-typical behavior (for example, grooming), which was not seen with CO_2_, suggesting a minimally negative animal experience and highlighting the potential of hypobaric hypoxia to offer a potential higher welfare alternative to CO_2_ exposure^44,45^. Although these behavioral data are encouraging, parallel neurophysiological measures are required to reliably determine the conscious period (where welfare state remains relevant), and to facilitate the interpretation of spontaneous behavior (for example, conscious motivated behavior rather than unconscious reflexive behavior).

Also relevant are the direct physical impacts of decompression on the animal, because exposure to reduced atmospheric pressure can cause gases within the body’s cavities to expand^52^. The consequences of this expansion vary according to the nature of the cavity and, more importantly, the rate at which decompression occurs^50,52–57^. Critically, when determining the effect of gradual decompression on animal welfare, we must consider the timing of potentially harmful hypobaric effects in relation to cognitive impairment, loss of consciousness or chamber recompression after death. Body cavities that ventilate passively (for example, sinuses, middle ear, lungs and so on) can equalize pressure changes if decompression rates are not rapid^52,58–60^. Nevertheless, there is the potential for some discomfort and/or pain during equalization of these cavities, as reported by human passengers during air travel^53^. Behavioral observations in pigs, mice and chickens show an increase in ventilation during hypobaric exposure and no evidence of breath-holding, suggesting a low risk of pulmonary injury under gradual decompression conditions, which is supported by pathological evidence from the same studies^45,57,61,62^. Importantly, human self-reported negative symptoms experienced during hypobaric hypoxia (as part of aviation training) include dysbarism of the ears and sinuses, trapped gas in the gastrointestinal tract and tooth pain but are predominantly experienced during recompression^63,64^. Our previous work on relatively fast decompression profiles (for example, equivalent ascent rate of 150 ms^−1^) show that these profiles were associated with pathological evidence of barotrauma (for example moderate middle ear congestion and hemorrhage) in all mice subjected to decompression; however, in the majority (~98%), the tympanic membranes remained intact^45^. Furthermore, we found no behavioral evidence indicative of pain or discomfort and could not determine when middle ear hemorrhage and congestion occured (for example, following loss of consciousness or during recompression, once the animal was dead)^44^.

The aim of this study was to evaluate EEG activity in mice exposed to gradual decompression and two CO_2_ exposure protocols (gas provided to the top or bottom of the chamber), with the aim of supporting a robust interpretation of spontaneous behavior during loss of consciousness. To do this, we carried out simultaneous EEG and behavioral monitoring to (1) characterize changes in EEG activity associated with different underlying mechanisms of brain death (that is, hypercapnic hypoxia (CO_2_) versus hypobaric hypoxia (via gradual decompression)), and (2) determine whether top filling the chamber with CO_2_ truly reflects best practice. Using these data, we draw conclusions on likely conscious experiences with each killing approach and determine whether gradual decompression offers true refinement over CO_2_.

## Results

**Fig. 1:**
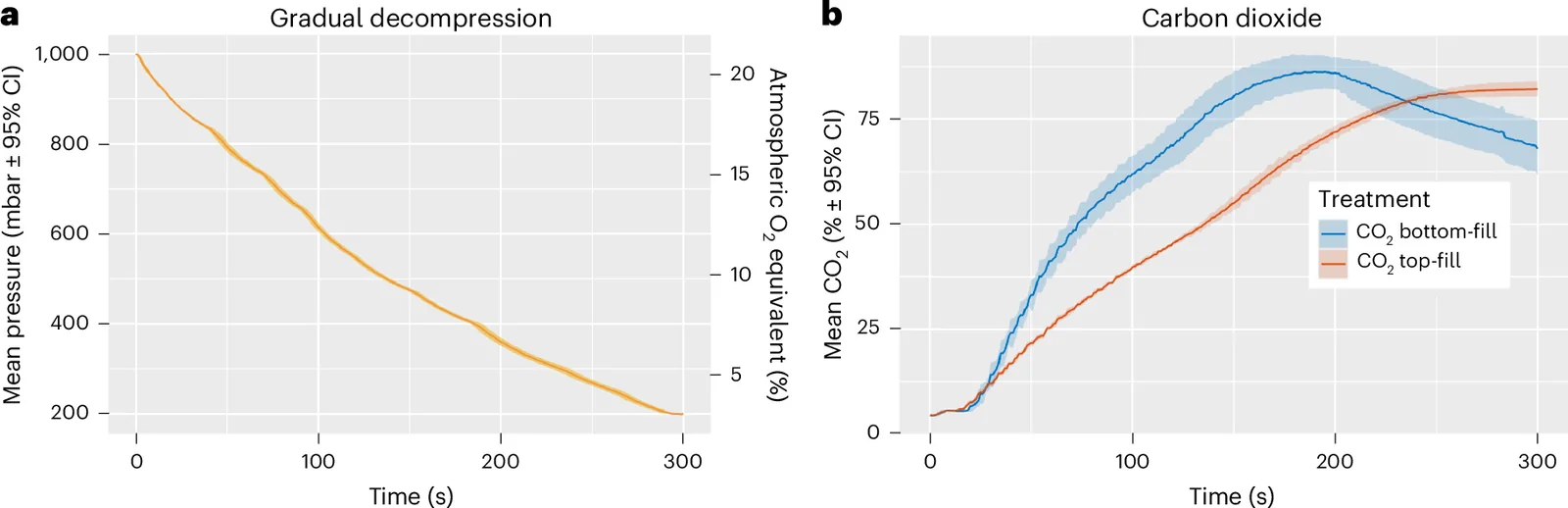
Terminal treatment profiles. **a**, Gradual decompression treatment profile: mean (±95% CIs) pressure (mbar) for mice undergoing gradual decompression during the first phase to reach the target pressure of 200 mbar (*n* = 8). This was followed by a 5-min hold period until death. The secondary axis represents calculated atmospheric oxygen equivalent (%) at sea level (1,013 mbar). **b**, Mean (±95% CIs) CO_2_ concentration (%) for CO_2_ profiles (*n* = 8 each); top (red) and bottom (blue) fill protocols.

All terminal treatments resulted in death of the mice, and none required early termination and emergency intervention. As outlined in Fig. 1a, gradual decompression at a slow linear rate (approximately 40 ms^−1^ ascension equivalent) resulted in a consistent reduction in mean atmospheric pressure until a target pressure of 200 mbar was achieved. Comparison of two CO_2_ exposure protocols (gas filled to the bottom or to the top of the chamber) were also conducted. Figure 1b reveals differences in the mean concentration of CO_2_ at mouse level according to the two exposure protocols. Filling the chamber with CO_2_ from the bottom results in higher concentrations of CO_2_ at mouse level earlier, compared with filling the chamber from the top.

### iEEG responses

**Fig. 2:**
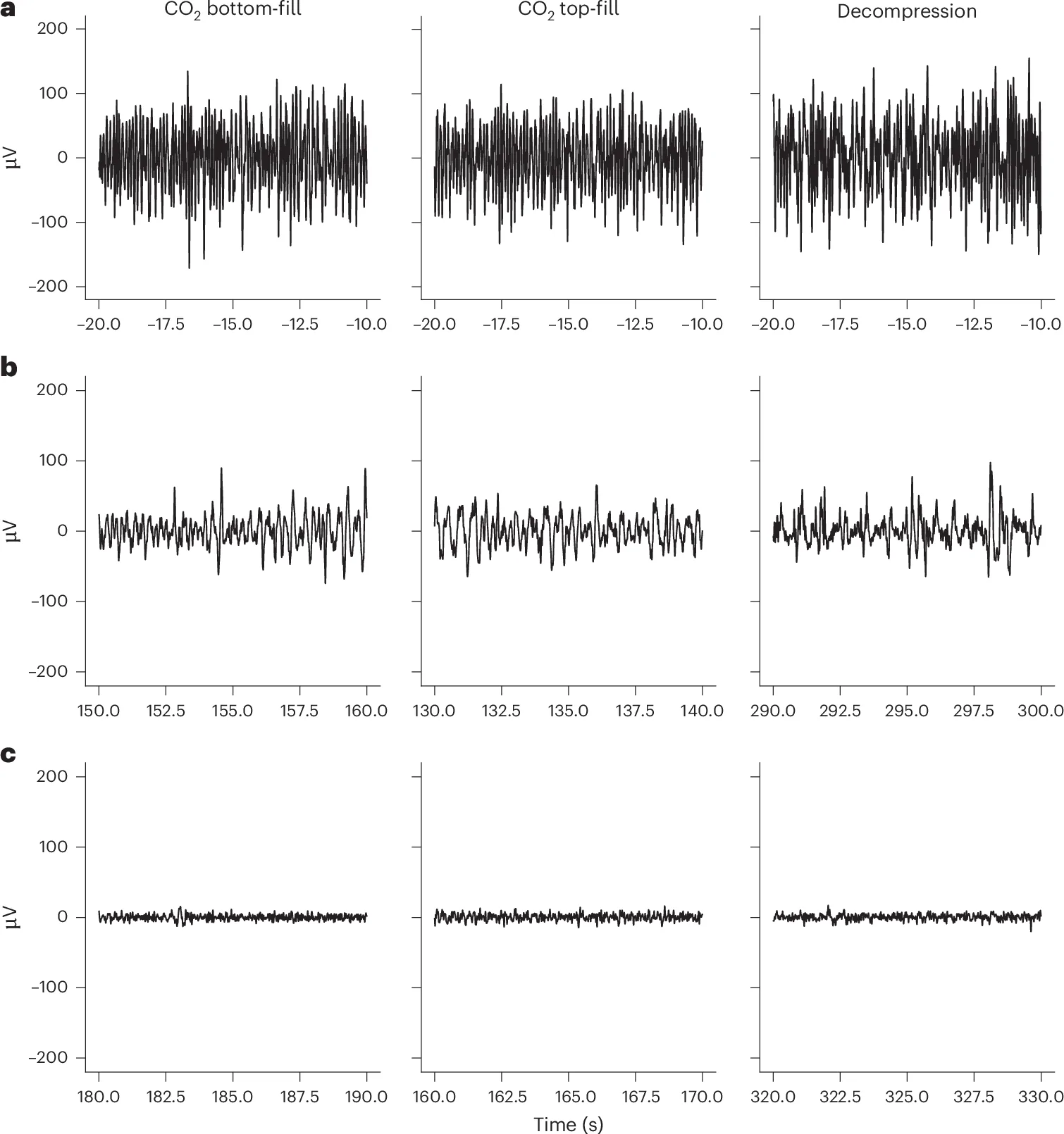
Representative iEEG traces. **a**–**c**, Representative iEEG trace excerpts (10 s duration) from mice undergoing hypobaric hypoxia (gradual decompression) and hypercapnic hypoxia via carbon dioxide gas delivered either to the top (CO_2_ top-fill) or the bottom (CO_2_ bottom-fill) of the chamber. For each treatment, **a**–**c** are from the same representative mouse (mouse IDs: 2.3, 1.7 and 1.8 for CO_2_ bottom-fill, CO_2_ top-fill and decompression, respectively). The traces illustrate the typical appearance of the iEEG during different states: while conscious during a 30 s baseline period (**a**); unconscious, with dominance of slow-wave (delta) activity, with the treatment application starting at time point 0 s (**b**); and following brain death, with remaining isoelectric activity (residual electrical noise of non-biological origin) (**c**). Please note the differing *x* axes (time in s) for each panel.

Intracranial EEG (iEEG) recordings were obtained from 22 mice undergoing one of three terminal treatments: gradual decompression (*n* = 7), CO_2_ top-fill (*n* = 8) or CO_2_ bottom-fill (*n* = 7). Recordings from two mice (one decompression and one CO_2_ bottom-fill) were excluded due to floating electrode artifact. Consistent changes in the visual appearance of the iEEG were apparent in all treatments (Fig. 2). During the baseline period when mice were fully conscious, iEEG activity consisted of high-frequency, low-amplitude waveforms as expected (Fig. 2a). During the application of each terminal treatment, the iEEG progressively transitioned from a high-frequency signal to a low-frequency signal (slow-wave activity; Fig. 2b), consistent with progressive transition to an unconscious state^8,9,11,14,65,66^. Finally, toward the end of each terminal treatment, the iEEG activity diminished to produce an isoelectric pattern (Fig. 2c), which consisted of residual low level nonbiological electric noise, reflecting a lack of neural activity and brain death.

**Fig. 3:**
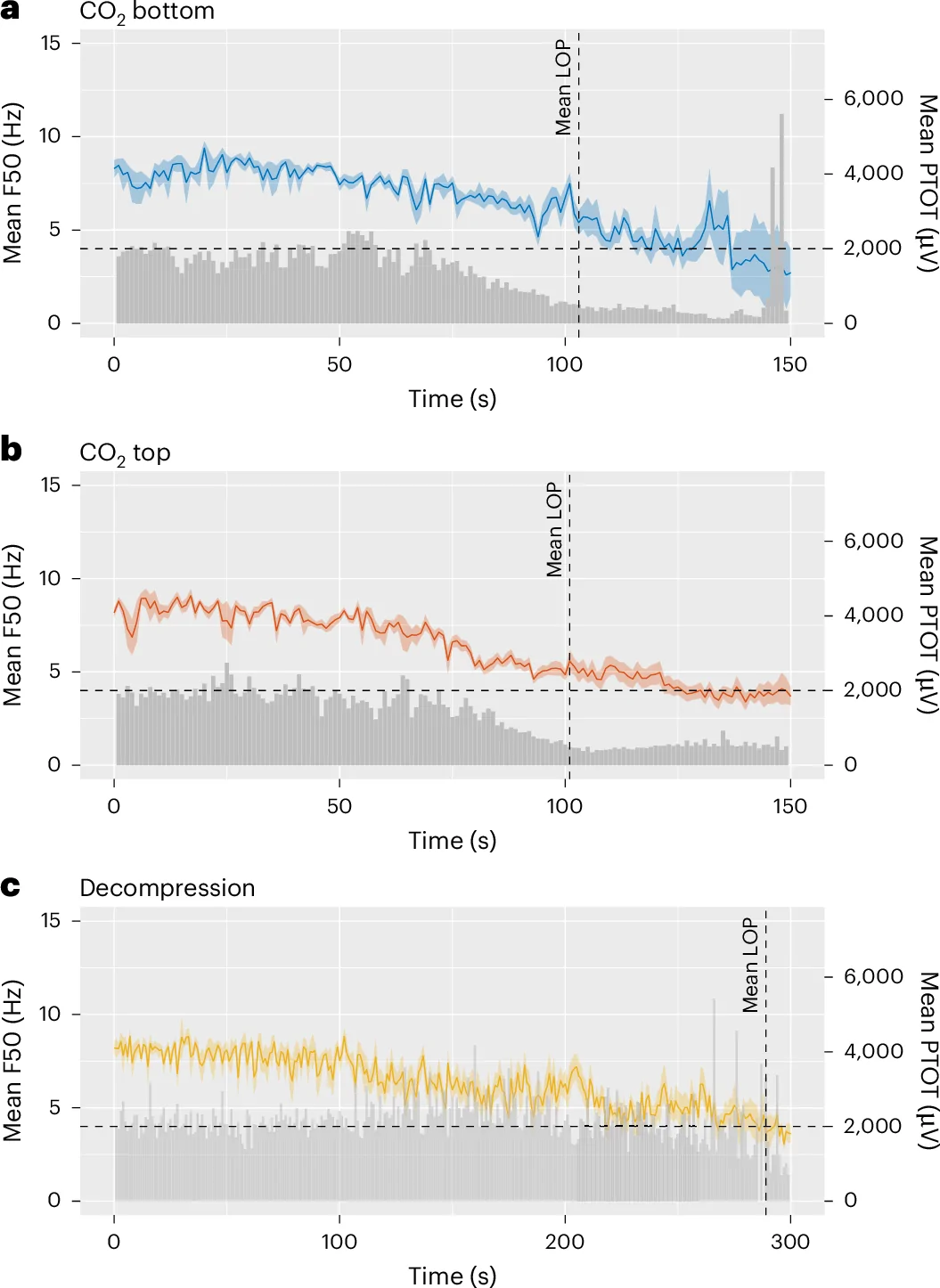
Effect of terminal treatments on F50 and PTOT. **a**–**c**, CO_2_ bottom-fill (*n* = 7) (**a**), CO_2_ top-fill (*n* = 8) (**b**) and gradual decompression (*n* = 7) (**c**). The *x* axis has been modified to reflect the different cycle lengths and therefore is limited to 150 s for CO_2_ top- and bottom-fill treatments (**a**,**b**) and 300 s for gradual decompression (**c**). The vertical dashed black line indicates the mean behavioral latency for LOP in each terminal treatment. The horizontal dashed black line indicates mean F50 equal to 4 Hz (an indicator of unconsciousness^10,67,94^). In **a**–**c**, mean ± s.e.m. F50 (Hz; line graph, left axis) and PTOT (μV; bar chart, right axis) are plotted over time in each terminal treatment.

As outlined in Fig. 3, the time point at which the iEEG waveform transitioned to slow (delta) wave (*X*^2^_(1,2)_ = 225.8, *P* < 0.0001) and isoelectric activity (*X*^2^_(1,2)_ = 581.5, *P* < 0.0001) depended upon the terminal treatment (estimated marginal mean ± standard error (s.e.) slow-wave activity: CO_2_ bottom-fill 120 ± 12.1 s, CO_2_ top-fill 117 ± 9.5 s, decompression 280 ± 8.9 s; mean ± s.e. isoelectric activity: CO_2_ bottom-fill 142 ± 10.4 s, CO_2_ top-fill 175 ± 10.3 s, decompression 359 ± 10.0 s). Importantly, owing to a longer cycle length, we observed increased latencies and durations of slow-wave (delta) activity in mice undergoing decompression compared to those exposed to CO_2_, irrespective of fill method (CO_2_ bottom-fill: *t*_15.5_ = 10.49, *P* < 0.001, CO_2_ top-fill: *t*_14.6_ = 12.42, *P* < 0.001), indicating that the transition to unconsciousness was more prolonged.

Traditional formal power spectrum analyses were carried out to quantitatively determine the spectral characteristics of the iEEG signal for each mouse. Using the power spectrum, total spectral power (PTOT) of the iEEG and median frequency (F50) were calculated for consecutive 1-s epochs during baseline and throughout terminal treatments. Increases in PTOT alongside decreases in F50 are well recognized in the literature as indicating loss of consciousness and anesthesia^8,9,11,14,65,66^. All mice were fully conscious and freely moving within the chamber during baseline, breathing atmospheric air, and there were no differences in baseline F50 between mice before undergoing terminal treatments (*X*^2^_(1,2)_ = 1.12, *P* = 0.552; CO_2_ bottom-fill: 7.8 ± 0.31 Hz, CO_2_ top-fill: 8.3 ± 0.31 Hz, decompression: 8.0 ± 0.32 Hz). There were also no differences in the baseline PTOT between mice (*X*^2^_(1,2)_ = 0.06, *P* = 0.969; CO_2_ bottom-fill: 1,849 ± 284 µV, CO_2_ top-fill: 1,943 ± 264 µV, decompression: 1,929 ± 402µV). F50 progressively declined upon application of each terminal treatment, indicating gradual loss of consciousness (*X*^2^_(1)_ = 902.54, *P* < 0.001; Fig. 3). Compared with both CO_2_ bottom-fill (*t*_4004_ = −8.22, *P* < 0.001, Fig. 3a) and CO_2_ top-fill (*t*_4004_ = −12.38, *P* < 0.001; Fig. 3b) this decline was more gradual with decompression (Fig. 3c); mean trends per 1-s interval: decompression = −0.01 Hz (95% confidence interval (CI) −0.011 to −0.009); CO_2_ top-fill = −0.029 Hz (95% CI −0.032 to −0.026); CO_2_ bottom-fill = −0.023 Hz (95% CI −0.026 to −0.020). We also observed differences between the two terminal CO_2_ treatments, whereby with bottom fill CO_2_ the decline in F50 was more gradual compared with top fill (*t*_4004_ = 2.79, *P* = 0.015).

Changes in PTOT were also evident, and depended upon terminal treatment (*X*^2^_(1,2)_ = 20.6, *P* < 0.001). Irrespective of the fill method, terminal treatment with CO_2_ resulted in progressive reduction in PTOT across time (mean trends per 1-s interval: CO_2_ bottom-fill (Fig. 3a) = −11.4 mV (95% CI −18.68 to −4.19); CO_2_ top-fill (Fig. 3b) = −15.0 mV (95% CI −21.97 to −8.07)). However, during decompression, PTOT showed a marginal increase at ~110 s alongside the marginal reduction in F50 (Fig. 3c).

**Fig. 4:**
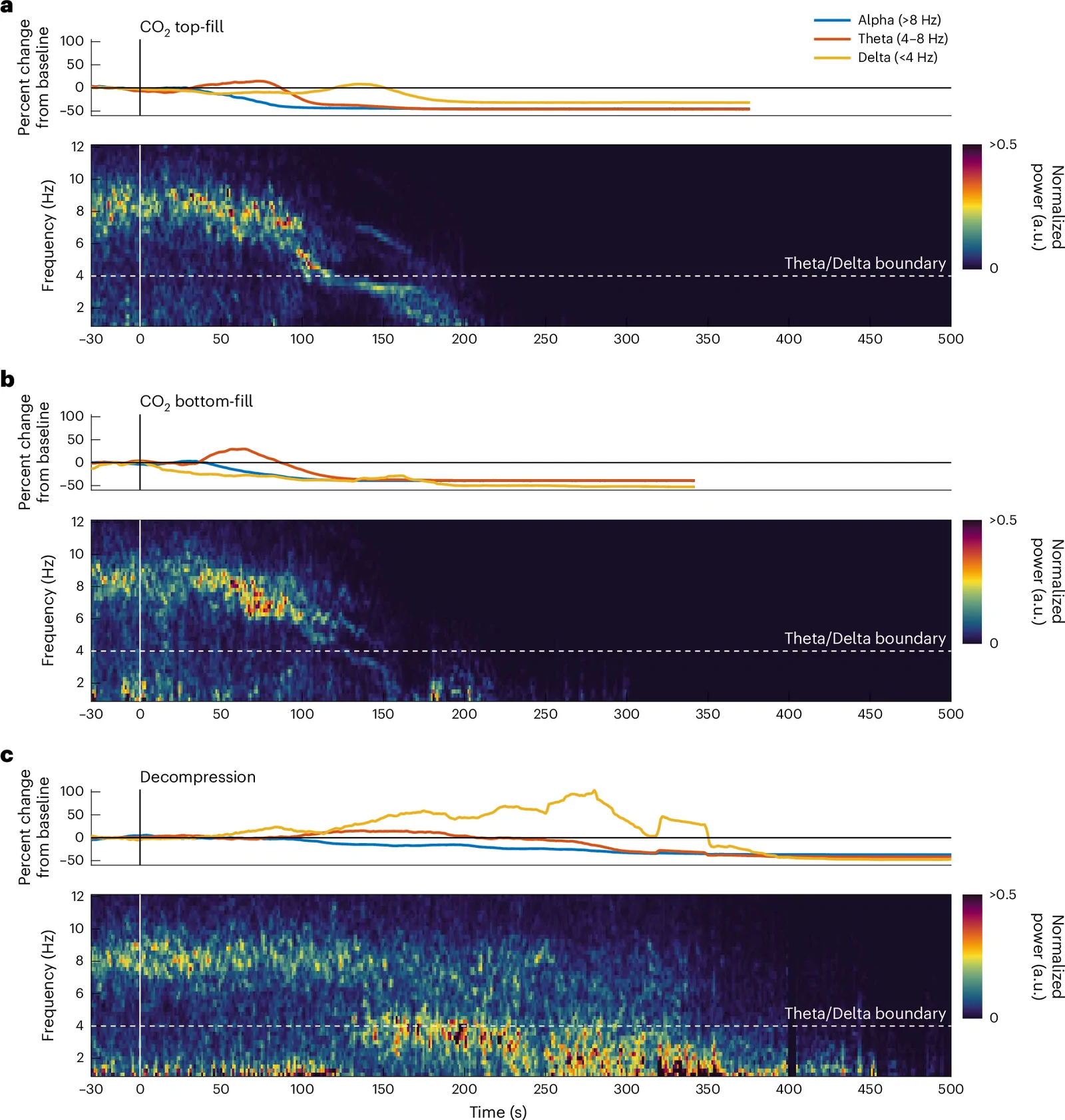
Time–frequency spectrograms for each terminal treatment. **a**–**c**, Average time–frequency spectograms (bottom color panels) of neuronal responses for mice undergoing terminal treatments: CO_2_ top-fill (*n* = 8) (**a**), CO_2_ bottom-fill (*n* = 7) (**b**) and gradual decompression (*n* = 7) (**c**). Normalized power is denoted by the intensity of the color according to the scale. Top line graphs denote the average percentage change from baseline in band power across alpha, theta and delta frequencies. Vertical solid white lines denote cycle/treatment start (0 s), and the horizontal dashed white lines denote the theta/delta frequency boundary at 4 Hz. a.u., arbitrary units.

Time–frequency analyses were conducted to better characterize the relative contribution (power) of each frequency band (alpha: 8–12 Hz, theta: 4–8 Hz, delta: <4 Hz) to the signal during each terminal treatment (Fig. 4). Time–frequency spectrograms (Fig. 4) highlight the changes in power in the frequency bands throughout each terminal cycle. Irrespective of terminal treatment, during the 30-s baseline period, the greatest power occurred in the alpha frequency band, centered around ~8 Hz. However, the temporal dynamics of the spectrogram differed according to terminal treatment. For CO_2_ top-fill (Fig. 4a), during the application of CO_2_, there is a progressive decrease in power in the alpha frequency band with power transitioning to the theta frequency band, peaking at ~50 s. The spectrogram highlights the increased power in the delta band, with peak power occurring at ~105 s (Fig. 4a). For CO_2_ bottom-fill (Fig. 4b), the spectrogram highlights a reduction in power in the alpha band until ~40 s, which is followed by an increase in power in the theta frequency band. However, unlike CO_2_ top-fill, there was no clear increase in delta activity during CO_2_ bottom-fill. For decompression (Fig. 4c), unlike both CO_2_ terminal treatments, we see a well-defined step change in power from alpha band activity (~8 Hz) to delta frequency band activity (<4 Hz) at ~105 s. These findings are in line with the marginal decrease in F50 and coincident increase in PTOT previously outlined (Fig. 3). This is clearly illustrated when examining the percentage change (from baseline) in delta band activity (Fig. 4, top). The greatest increase in power occurs in the delta band during decompression, with a percentage increase above baseline power peaking at 80%, occurring between ~105 s and ~300 s into the cycle.

### Heart rate responses

We obtained electrocardiogram (ECG) waveforms with clean QRS complexes for 20 mice undergoing terminal treatments (decompression *n* = 6; CO_2_ bottom-fill *n* = 6; CO_2_ top-fill *n* = 8). Data from four mice were excluded due to poor-quality recordings with no detectable QRS complexes. There were no differences in the mean baseline heart rates between mice before undergoing terminal treatments (*X*^2^_(1,2)_ = 0.21, *P* = 0.901; CO_2_ bottom-fill: 740 ± 15.4, CO_2_ top-fill: 733 ± 14.1, decompression: 730 ± 15.2 bpm). Both hypercapnic hypoxia (CO_2_ top and bottom filled) and hypobaric hypoxia were associated with progressive declines in heart rate (hypoxic bradycardia) over time. There was no evidence of increased heart rate for any terminal treatment. The steepest heart rate decline occurred during exposure to CO_2_ bottom-fill (mean trends per 1-s interval = −2.52 bpm (95% CI –2.79 to −2.26)), followed by CO_2_ top-fill (mean trends per 1-s interval = −2.37 bpm (95% CI –2.63 to −2.11)), with decompression resulting in a more gradual reduction in heart rate (mean trends per 1-s interval = −1.44 bpm (95% CI –1.56 to −1.31)) (Supplementary Fig. 1). As such, terminal treatment impacted the latency to bradycardia (defined here as a 30% reduction from baseline heart rate^11^) (*X*^2^_(1,2)_ = 78.3, *P* < 0.0001). Decompression resulted in longer latencies (mean: 214 ± 8.3 s) to bradycardia than both CO_2_ top-fill (mean: 167 ± 7.9 s) and CO_2_ bottom-fill (mean: 124 ± 9.1 s) treatments (*t*_15_ = 4.14, *P* = 0.002; *t*_10.9_ = 7.6, *P* < 0.001, respectively). Furthermore, CO_2_ bottom-fill resulted in earlier bradycardia than CO_2_ filled via the top (*t*_15_ = 4.14, *P* = 0.002).

### Behavioral responses

Extensive behavioral assessment was conducted throughout each terminal treatment. As outlined in Table 1, mice undergoing decompression had longer latencies to LOP (*X*^2^_(2)_ = 945.77, *P* < 0.001), ataxia (*X*^2^_(2)_ = 223.67, *P* < 0.001), motionless (*X*^2^_(2)_ = 314.93, *P* < 0.001), abnormal breathing (*X*^2^_(2)_ = 93.95, *P* < 0.001), first gasp (*X*^2^_(2)_ = 49.19, *P* < 0.001), first agonal gasp (*X*^2^_(2)_ = 321.61, *P* < 0.001), last agonal gasp (*X*^2^_(2)_ = 223.64, *P* < 0.001) and apnea (*X*^2^_(2)_ = 329.69, *P* < 0.001) compared with CO_2_ treatments (Table 1). Top-fill CO_2_ resulted in longer latencies to motionless (*t* = 3.298, *P* = 0.021), agonal gasping (*t* = 3.580, *P* = 0.001), last agonal gasp (*t* = 4.470, *P* < 0.001) and apnea (*t* = 3.647, *P* < 0.001) compared with CO_2_ supplied to the bottom of the chamber (Table 1).

**Table 1:** Summary of behavioral latencies by terminal treatment

Behavioral latencies	CO_2_ bottom-fill (mean ± s.e.) (s) (*n*)	CO_2_ top-fill (mean ± s.e.) (s) (*n*)	Decompression (mean ± s.e.) (s) (*n*)
LOP	104 ± 3.71^a^ (*n* = 8)	102 ± 3.67^a^ (*n* = 8)	290 ± 6.44^b^ (*n* = 8)
Ataxia	72.9 ± 4.55^a^ (*n* = 8)	63 ± 4.01^a^ (*n* = 8)	170.3 ± 8.87^b^ (*n* = 8)
Motionless	176 ± 11.0^a^ (*n* = 8)	227 ± 11.1^b^ (*n* = 8)	368 ± 11.7^c^ (*n* = 8)
Abnormal breathing	40 ± 10.97^a^ (*n* = 8)	32.9 ± 9.79^a^ (*n* = 8)	127.7 ± 9.87^b^ (*n* = 8)
First gasp	79.8 ± 12.2^a^ (*n* = 8)	67.2 ± 12.1^a^ (*n* = 8)	154.7 ± 12.8^b^ (*n* = 8)
First agonal gasp	137 ± 5.52^a^ (*n* = 8)	167 ± 6.63^b^ (*n* = 8)	311 ± 10.72^c^ (*n* = 8)
Last agonal gasp	163 ± 7.29^a^ (*n* = 7)	220 ± 10.7^b^ (*n* = 5)	365 ± 13.62^c^ (*n* = 7)
Apnea	135 ± 5.98^a^ (*n* = 8)	166 ± 6.81^b^ (*n* = 8)	315 ± 11.96^c^ (*n* = 7)
Dig	4.3 ± 5.09^a^ (*n* = 1)	14.8 ± 7.34^a^ (*n* = 5)	31.4 ± 12.99^a^ (*n* = 7)
Interrupted grooming bout	6.77 ± 3.12^a^ (*n* = 6)	6.80 ± 4.74^a^ (*n* = 3)	7.79 ± 3.38^a^ (*n* = 7)
Grooming bout	4.62 ± 3.2^a^ (*n* = 4)	*	43.69 ± 25.6^b^ (*n* = 5)
Rear	13 ± 7.8^a^ (*n* = 8)	12.3 ± 6.96^a^ (*n* = 8)	19.2 ± 7.02^a^ (*n* = 8)
Head up	13.75 ± 3.78^a^ (*n* = 8)	5.59 ± 3.29^a^ (*n* = 8)	14.41 ± 3.46^a^ (*n* = 7)

Mean ± standard error (s.e.) of behavioral latencies according to terminal treatment: hypobaric hypoxia (decompression) and hypercapnic hypoxia via carbon dioxide gas either from the top (CO_2_ top-fill) or from the bottom (CO_2_ bottom-fill) of the chamber. Reported values are estimated marginal means from statistical modeling. Different letters denote a statistical difference between terminal treatments in a behavioral latency for a specified behavior in each row (generalized linear mixed-effects models, *P* < 0.05). An asterisk identifies a behavior that was not performed in a terminal treatment.

We also investigated total counts of behavioral events because these provide meaningful measures relating to total harm during the conscious period. Mice exposed to CO_2_ gasped more than mice exposed to decompression (*X*^2^_(2)_ = 39.05, *P* < 0.001), irrespective of fill method (*Z*_ratio _= 5.12, *P* < 0.001, *Z*_ratio_ = 5.78, *P* < 0.001, respectively). Mice undergoing decompression performed more rears compared with CO_2_ top-fill (*Z*_ratio _= 3.83, *P* = 0.0004), but not CO_2_ bottom-fill (*Z*_ratio _= 1.97, *P* = 0.120), and performed more digging events compared with both CO_2_ terminal treatments (top: *Z*_ratio_ = 4.62, *P* < 0.001; bottom: *Z*_ratio_ = 4.25, *P* = 0.0001). We also found that bottom-filled CO_2_ resulted in fewer agonal gasps than both decompression (*Z*_ratio _= 2.51, *P* = 0.030) and top-filled CO_2_ (*Z*_ratio _= 3.40, *P* = 0.002). We found no effect of terminal treatment on the number of other behavioral events performed, including escape attempts, ear scratches, grooming bouts, interrupted grooming bouts, head shakes, head flicks or the number of head up postures (Table 2).

**Table 2:** Summary of behavioral counts by terminal treatment

Behavioral counts	CO_2_ bottom-fill (mean ± s.e.) (*n*)	CO_2_ top-fill (mean ± s.e.) (*n*)	Decompression (mean ± s.e.) (*n*)
Gasp	40.9 ± 4.76^a^ (*n* = 8)	37.8 ± 4.31^a^ (*n* = 8)	19.1 ± 2.37^b^ (*n* = 8)
Agonal gasp	12.6 ± 2.94^a^ (*n* = 8)	37.4 ± 8.17^b^ (*n* = 8)	28.2 ± 6.24^b^ (*n* = 8)
Escape attempt	2.25 ± 1.38^a^ (*n* = 8)	1.125 ± 0.74^a^ (*n* = 8)	0.125 ± 0.144^a^ (*n* = 8)
Ear scratch	0 ± 0.00^a^ (*n* = 8)	0 ± 0.00^a^ (*n* = 8)	1.5 ± 1.18^a^ (*n* = 8)
Rear	9.19 ± 1.73^a,b^ (*n* = 8)	5.65 ± 1.15^a^ (*n* = 8)	14.28 ± 2.47^b^ (*n* = 8)
Grooming bout	0.5 ± 0.25^a^ (*n* = 8)	0 ± 0.00^a^ (*n* = 8)	0.75 ± 0.306^a^ (*n* = 8)
Interrupted grooming bout	1.66 ± 0.76^a^ (*n* = 8)	0.87 ± 0.51^a^ (*n* = 8)	3.21 ± 1.31^a^ (*n* = 8)
Head shake	0.0991 ± 0.115^a^ (*n* = 8)	0.0983 ± 0.117^a^ (*n* = 8)	0.3656 ± 0.349^a^ (*n* = 8)
Head flick	0.0983 ± 0.107^a^ (*n* = 8)	0 ± 0.00^a^ (*n* = 8)	0.7261 ± 0.477^a^ (*n* = 8)
Head up	1.662 ± 0.758^a^ (*n* = 8)	0.868 ± 0.505^a^ (*n* = 8)	3.205 ± 1.306^a^ (*n* = 8)
Dig	0.226 ± 0.177^a^ (*n* = 8)	0.214 ± 0.164^a^ (*n* = 8)	3.733 ± 2.386^b^ (*n* = 8)

Mean ± standard error (s.e.) of behavioral counts according to terminal treatment: hypobaric hypoxia (decompression) and hypercapnic hypoxia via carbon dioxide gas either from the top (CO_2_ top-fill) or from the bottom (CO_2_ bottom-fill) of the chamber. Reported values are estimated marginal means from statistical modeling. Different letters denote a statistical difference between terminal treatments in a behavioral count for a specified behavior in each row (generalized linear mixed-effects models, *P* < 0.05).

Several behaviors were performed infrequently, and not all mice performed all behaviors; therefore, we determined the likelihood of mice performing these behaviors to provide an indication of a potentially negative animal experience during the conscious period. The only behavior where terminal treatment affected the likelihood of expression was ear scratching (*X*^2^_(2)_ = 13.98, *P* < 0.001), where mice undergoing decompression were more likely to ear scratch than mice exposed to CO_2_ (Table 3). We found no effect of terminal treatment on the likelihood of performing escape attempts, head shakes or head flicks (Table 3).

**Table 3:** Number and percentage of individuals exhibiting infrequent behaviors

Behavior	CO_2_ top-fill (*n*, %)	CO_2_ bottom-fill (*n*, %)	Decompression (*n*, %)
Escape attempts	3/8 (37.5%)^a^	4/8 (50%)^a^	1/8 (12.5%)^a^
Ear scratches	0/8 (0%)^a^	0/8 (0%)^a^	5/8 (62.5%)^b^
Head shakes	1/8 (12.5%)^a^	1/8 (12.5%)^a^	3/8 (37.5%)^a^
Head flicks	0/8 (0%)^a^	1/8 (12.5%)^a^	4/8 (50%)^a^

Number (*n*) and percentage (%) of mice performing infrequent behaviors observed, according to terminal treatment: hypobaric hypoxia (decompression) and hypercapnic hypoxia via carbon dioxide. Different letters denote a statistical difference between terminal treatments in absolute behavioral occurrence per individual mouse for a specified behavior in each row (binary logistic regression models, *P* < 0.05).

**Fig. 5:**
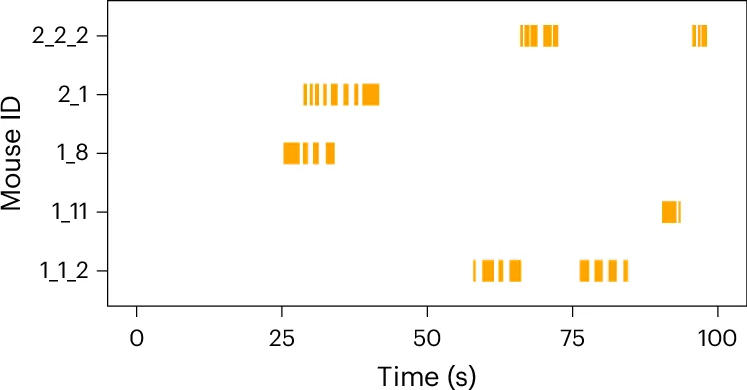
Ear scratching during gradual decompression. Individual ear scratching events (s) for mice performing ear scratching (*n* = 5/8) while undergoing gradual decompression.

Five out of eight mice (62.5%) undergoing decompression performed ear scratches early in the cycle and during the conscious period (Fig. 5).

### Integrated findings

**Fig. 6:**
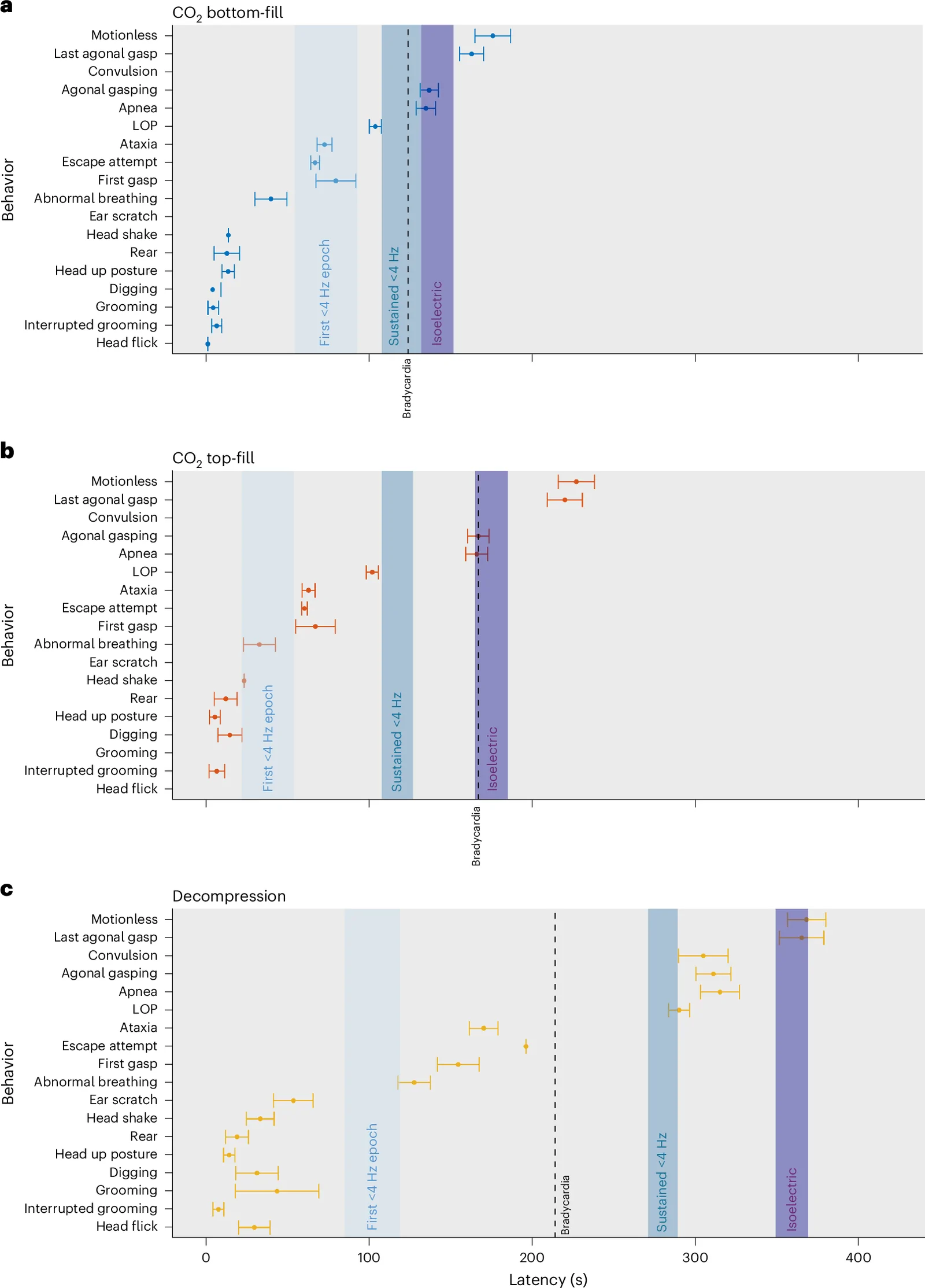
Order of behavioral and neurophysiological events. **a**–**c**, Mean ± standard error (s.e.) latencies of behavioral and neurophysiological events according to each terminal treatment CO_2_ bottom-fill (*n* = 7) (**a**), CO_2_ top-fill (*n* = 8) (**b**) and gradual decompression (*n* = 7) (**c**). Ribbons correspond to latencies (mean ± s.e.) of key traditional spectral characteristics (for example, based on F50 and PTOT values). The light-blue ribbon indicates the first 1-s epoch where the F50 is <4 Hz, the mid-blue ribbon indicates sustained epochs where the F50 is <4 Hz, and the dark-blue ribbon indicates isoelectric activity. The dashed line denotes the latency for bradycardia (≥30% reduction from baseline heart rate).

Combining simultaneous neurophysiological, cardiovascular and behavioral measures allows a better understanding of what the animals probably experience before loss of consciousness. Figure 6 illustrates the sequence of behavioral events mapped against key spectral variables (F50) from our power spectrum analyses. This illustrates the difference in cycle length between gradual decompression and the two CO_2_ treatments, and highlights that the sequence of behavioral events is conserved across treatments. A critical difference to highlight between decompression and CO_2_ treatments is the potential reliability of LOP as a behavioral indicator of loss of consciousness. For CO_2_ treatments, the summarized latencies for LOP occurred before those for sustained iEEG epochs characterized with F50 <4 Hz, while in the decompression treatment it either occurred alongside or immediately after the onset of iEEG defined loss of consciousness (that is, equivalent to general anesthetic, sustained F50 <4 Hz). Figure 3 further demonstrates these findings.

During decompression, slow waveforms were dominant from ~105 s, after which changes in respiratory behaviors (abnormal breathing and first gasp), ataxia and LOP occured (in addition to the one escape attempt performed by a single mouse undergoing decompression). In mice exposed to CO_2_ (irrespective of fill method), respiratory behaviors, ataxia and escape attempts all occurred before LOP and before the iEEG had transitioned to slow-wave (<4 Hz) activity and therefore could be indicative of potential negatively valenced sensory–affective experiences during a semi-conscious phase. Furthermore, during CO_2_ bottom-fill, bradycardia did not occur until mice were unconscious (that is, during sustained <4 Hz delta activity), while for CO_2_ top-fill, bradycardia occurred later, when the iEEG was isoelectric. By contrast, during decompression, bradycardia occured during the period dominated by slow-wave activity, and occurred ~76 s before LOP.

Lastly, assessment and occurence of bilateral tympanic membrane rupture was conducted in all mice for all three treatments, after confirmation of death. All mice showed no evidence of either bilateral or unilateral tympanic membrane rupture.

## Discussion

Hypobaric hypoxia (via gradual decompression) resulted in slow-wave dominance substantially before the occurrence of overt respiratory behaviors and before behavioral LOP. These findings support the notion of early cognitive impairment and an impaired state of consciousness and could reflect reduced awareness of respiratory distress such as breathlessness and air hunger. This is contrary to our findings from mice undergoing hypercapnic hypoxia (via exposure to CO_2_ gas) where respiratory behaviors, escape attempts and LOP occurred before the onset of slow-wave activity, when mice are likely to retain the capacity for negatively valenced sensory–affective experiences associated with hypercapnia.

Combining traditional formal power spectral analyses with time–frequency analyses revealed the spectral characteristics and temporal dynamics of the iEEG signal and uncovered the relative changes in power across time in key frequency bands (alpha: 8–12 Hz, theta: 4–8 Hz, delta: <4 Hz). The transition from a conscious to an unconscious state is generally described to occur when peak power transitions from higher frequency bands to the lower delta frequency band (commonly referred to as slow-wave activity)^9,11,14,65^. This transition of peak power to the lower delta frequency band, defined as <4 Hz, has been extensively considered to be an indicator of absence of consciousness in laboratory mice during deep sleep^10^ and anesthesia^67^. For mice undergoing decompression, we show that slow-wave activity (<4 Hz) was dominant early in the cycle (~105 s) despite the animal not losing posture (a behavioral proxy for loss of consciousness) until much later (~290 s). Changes in the visual and quantitative characteristics of EEG due to hypoxia have been studied since the 1930s^68,69^. Consistent features in human participants exposed to hypobaric hypoxia, such as those used to simulate high-altitude flight in military aviation, include a transition toward increased delta activity and a decrease in alpha activity over acute periods (minutes)^47^. This is a consequence of a metabolic insult impairing brain function causing impairment in cognitive, visual, psychomotor and psychological domains^47^. As such, signs and symptoms in humans range from an inability to perform simple tasks and impaired night vision, to more severe symptoms such as, impaired neuromuscular control, sensory loss and moderate-to-severe cognitive impairment^47,68^. It is generally accepted that moderate-to-severe impairment occurs at altitudes above 15,000 ft (4,472 m)^47,48^, whereby an individual’s cognitive capabilities become increasingly compromised until they lose consciousness^48,70^. In the present study, the pressure in the hypobaric chamber at 105 s (when slow-wave activity becomes dominant) was ~600 mbar (60 kPa), which is equivalent to an altitude of ~14,108 ft (~4,300 m). Therefore, given the comparable altitude equivalent to humans reporting moderate-to-severe impairment combined with the onset of slow-wave activity, it is reasonable to assume that mice would also have reduced brain function and cognitive impairment during the early stages of the decompression cycle (from ~105 s). If so, this could result in an impediment to their ability to detect and fully experience any negative effects of decompression before losing consciousness. However, given an animal’s inability to self-report, it remains unknown whether and when symptoms transition from mild to severe impairment and the extent to which mice can experience affective state during this transitory phase. Future research could use operant behavioral paradigms to further elucidate the affective states experienced by animals. Such approaches can reveal animals’ preferences or aversions to specific stimuli and how the strength of these responses’ influences perception, motivation and learning in relevant contexts (for example, killing scenarios)^71–73^.

Hypercapnic hypoxia results in hypoxia through a different mechanism, that is, an influx of an alternative gas (CO_2_) rather than due to changes in the partial pressure of oxygen. However, hypercapnic hypoxia also results in increased slow-wave activity in EEG in humans^74^. Whether this is a consequence of hypercapnia and/or hypoxia alone or in combination remains unknown. Exposure to carbon dioxide is considered a useful short-acting anesthetic because it induces rapid acidosis in the cerebrospinal fluid^75^. Therefore, despite producing EEG changes similar to those seen with hypobaric hypoxia, the underlying mechanisms are different. Nonetheless, when assessing the welfare implications of each method, it is important to evaluate when this transition to increased slow-wave activity occurs relative to changes in spontaneous behavior. When exposed to hypercapnic hypoxia, the transition of power from higher- to lower-frequency bands occurred after the animal had lost posture; with power in higher-frequency bands maintained throughout the gas cycle. This suggests that the animals may retain some capacity to experience negatively valenced sensory–affective states associated with hypercapnia^29,33,68,76,77^ up until, or even after they have lost posture. This finding is particularly concerning when considering existing protocols for manually operated CO_2_ systems^29,32,34^, which often rely on the operator increasing the flow rate—potentially to levels above those associated with nociception—when LOP is observed.

Behavioral assessment is critical for assessing an animal’s underlying affective (emotional) state and making inferences with regard to their welfare^78–80^. In line with our previous work^44^, we took a mechanistic approach to ethogram design, avoiding assigning motivational drive to observed behaviors. This was further enhanced by combining neurophysiological data to better inform whether a behavior is likely conscious or unconscious in nature. Dyspnea (breathlessness) reflects altered respiratory function and/or activation of respiratory reflexes and can elicit negative affective experiences in humans (for example anxiety, fear and panic associated with an urge to breath)^28^. We reported more gasping (defined as oral breathing whereby the mouse opens its mouth and holds the mouth open to breath) in mice exposed to CO_2_ than in mice exposed to gradual decompression^44^. Notably, gasping occurred before slow-wave activity, thus probably during the conscious phase. Consequently, it is possible that mice experience respiratory distress including breathlessness and air hunger^28^. This contrasts with gradual decompression during which abnormal breathing and first gasp are occurring during slow-wave dominance when mice are likely to have substantially impaired brain function associated with reduced ‘conscious’ awareness of feelings relating to respiratory distress, including air hunger.

As previously highlighted^29,44^, hypercapnic stimulation of dyspnea may also underpin the greater number of escape attempts observed during exposure to CO_2._ Vertical jumping is a well-documented behavioral response in mice exposed to CO_2_ (ref. ^81^). Owing to CO_2_ being heavier than air, it is possible that the way the gas is introduced to the chamber influences the concentration of the gas at the mouse level and therefore the associated welfare consequences. We compared two methods of gas delivery (top and bottom fill), and the top-fill methodology resulted in fewer vertical jumps than have previously been reported in the literature^32,44,82–86^. Top-fill methodologies are suggested to avoid exposure to high levels of CO_2_ at mouse level by facilitating a more even distribution of gas throughout the chamber. However, so far there are no direct scientific data supporting this hypothesis, which perhaps explains why we recently found that most UK-based institutions still utilize bottom-fill approaches, frequently using modified anesthetic chambers^21,29^. Our findings demonstrate that, despite the use of the same CO_2_ flow rate (20% of the chamber volume per minute) for both top- and bottom-fill CO_2_ methods, the CO_2_ concentration at mouse level was substantially higher with bottom-fill delivery. This observation emphasizes that identical flow rates can create different fill patterns in the chamber according to the properties of the gas and how the chamber is filled. Exposure to CO_2_ is known to be nociceptive above 40%^29,82,84,87^; therefore, we demonstrate that it is possible that bottom-fill approaches result in nociception and pain even at recommended fill rates (for example, 10–30% in the UK^34^ or 30–70% of chamber volume per minute in the USA^40^), resulting in unnecessary suffering during euthanasia. It is essential to not only specify the flow rate, but also the filling method and the need for accurate monitoring of gas concentrations at animal level, to ensure that the procedure is applied correctly to avoid unnecessary suffering. Despite the evidence for the negative affective consequences of CO_2_, we present the first direct evidence supporting the guidance on utilizing top-fill approaches for CO_2_ and urge institutions utilizing bottom-fill approaches to refine their practices and adopt top-fill approaches.

Furthermore, our results highlight the potential limitations of relying solely on behavioral indicators for loss of consciousness (for example, LOP). LOP occurred before the onset of sustained iEEG epochs, characterized with F50 <4 Hz (that is, equivalent to general anesthesia), for both CO_2_ treatments. By contrast, for decompression, LOP either occurred alongside or immediately after the onset of iEEG-defined loss of consciousness. Although the interval between LOP and iEEG indicators of loss of consciousness during CO_2_ exposure was relatively short (approximately 10–20 s), it may potentially have substantial animal welfare consequences. In line with current best practice guidelines in the UK and Europe^32,34^, CO_2_ gas flow was maintained at 20% volume per minute until LOP was observed, at which point it was increased to 80% of the chamber volume per minute. Therefore, it is possible that mice were not fully unconscious (based on iEEG characteristics) and thus may have been subjected to unnecessary suffering due to exposure to aversive concentrations of CO_2_ (for example, top-fill: ~38% CO_2_ at mean LOP; bottom-fill: ~63% CO_2_ at mean LOP). Therefore, we suggest a refinement to current best practice recommendations, where LOP is redefined as an early indicator of onset of unconsciousness (that is, a precursor) and, subsequently, the CO_2_ flow rate should not be increased at the point of LOP and should instead be instigated 10–20 s after LOP onset, to minimize the risk of sedated mice (that is, not fully unconscious) being unnecessarily subjected to aversive CO_2_ concentrations.

The primary concern arising from our previous work assessing the implications of gradual decompression is the possibility for pain and/or discomfort arising from barotrauma in the middle/inner ear^44,45^. Despite the tympanic membrane being intact in 100% of mice, we here again found evidence of moderate haemorrhage of the tympanic bulla in all mice undergoing decompression. Consequently, in accordance with previous work^44,45^, we focused our attention on head- and ear-related behaviors during the conscious phase. Mice were more likely to ear scratch during the conscious phase when undergoing decompression compared with CO_2_. However, in accordance with previous findings^44^, not all mice undergoing decompression performed ear scratching (*n* = 5/8, 62.5%) and they performed species-typical behaviors (for example grooming and digging) during the conscious period, which is encouraging form a welfare perspective. Previous work found no effect of analgesia on the likelihood of ear scratching, and it also occurred under sham conditions^44^. Thus, while we cannot rule out the possibility that ear scratching does reflect pain and/or discomfort, we have no specific evidence to support this interpretation. The presence of exploratory and maintenance behaviors is noteworthy and supports the notion that gradual decompression constitutes a minimally negative experience for mice. Taken together with other behavioral signs, it is possible that the observed pathological changes may be painless or are occurring after loss of consciousness. In addition, we cannot rule out the possibility that these pathological changes occur during recompression (descent from altitude), not decompression (ascent to altitude). Nonetheless, these issues could be further investigated in future work directly assessing cognitive capabilities and degree of aversion during the conscious phase in mice undergoing decompression.

Combining neurophysiological data with behavioral assessment provides a more comprehensive evaluation of animal welfare by utilizing a direct and objective measure for determining the period during which animals remain conscious, which is the period of concern for animal welfare. Our findings succinctly demonstrate that behavioral proxies, such as LOP, do not always directly relate to neurophysiological data and are highly dependent upon the underlying mechanism of action^76,77,88–91^. While LOP was a conservative measure of loss of consciousness in mice undergoing hypobaric hypoxia (gradual decompression), it was unreliable when considering the onset of unconsciousness during hypercapnic hypoxia (CO_2_). Specifically, neurophysiological data did not support the onset of an unconscious state (defined as F50 <4 Hz in laboratory mice^12,67^) until after the mice had lost posture when exposed to CO_2_, which raises key questions surrounding their sensory capacity to experience pain and negative affective states while recumbent. Furthermore, the utility of physiological changes in ECG activity and heart rate as standalone indicators of welfare states may be limited in terminal treatments, as treatment-induced physiological insults might make these parameters less suitable to detect the presence of negative affective states such as fear and panic (that is, both terminal treatments cause bradycardia, which may mask tachycardia).

We adopted time–frequency analyses used in neuroscience, thus providing a novel approach in this context (welfare assessment at killing). The novel implementation of time–frequency-resolved iEEG (0.25 Hz frequency resolution) during hypobaric hypoxia and hypercapnic hypoxia allowed us to gain greater insight into the intricate changes in power across neuronal frequencies during the transition from a conscious to an unconscious state. This approach was complementary to the more traditional analyses used previously in this context; assessing changes in F50 and PTOT^9,11^. Although widely used, standard spectral analysis approaches are based on global averages of EEG signal (that is, average F50 and average PTOT) at a given time point (for example, 1-s epochs). Consequently, such measures are more susceptible to noise artifacts and are unable to pinpoint temporal changes in each of the frequency bands simultaneously. Nonetheless, these measures also highlighted the different mode of action on brain activity during hypobaric hypoxia and hypercapnic hypoxia. During the transition from a conscious to an unconscious state, PTOT typically increases while F50 decreases^9^, which was apparent from ~110 s in mice undergoing hypobaric hypoxia, corroborating our findings from the time–frequency spectrograms. These findings suggest the progressive dominance of slow-wave, high-amplitude EEG activity, which has a lower mean F50 than EEG wavefrom activity associated with consciousness^8,11,65,89,91^. However, in mice exposed to CO_2_, we did not observe the marginal increase in PTOT alongside the reduction in F50, which is perhaps due to the anesthetic properties of CO_2_ (ref. ^75^).

Although the data from this study remain encouraging, they have several limitations. Sample sizes were relatively small, and experiments were conducted in a single mouse strain using only male animals, which may limit the generalizability of the findings. Future studies should assess whether these findings are consistent across additional mouse strains and in both sexes. Although sex differences cannot be excluded on the basis of the data presented here, global physiological insult produced by hypobaric hypoxia, together with the available literature^92,93^, suggests that sex-dependent differences are unlikely. Finally, although the gradual decompression cycle investigated here takes longer than killing using CO_2_ (594 s versus 332 s), the additional time should not preclude its use if it results in improved animal welfare outcomes.

## Conclusion

Hypobaric hypoxia (gradual decompression) results in a more gradual transition toward unconsciousness in laboratory mice; however, brain activity data suggest that the onset of cognitive impairment and emergence of anesthetic-like brain states occur before LOP. While some concern surrounding potentially negative experiences due to barotrauma remain, the behavioral data are encouraging. By contrast, hypercapnic hypoxia (exposure to CO_2_) results in a more rapid transition toward unconsciousness in laboratory mice, but our data support longstanding concerns surrounding an animal’s capacity to experience pain, anxiety and breathlessness, before and possibly even after LOP. Notwithstanding the urgency to move away from CO_2_ exposure entirely, we emphasize the necessity for research institutions to revise existing CO_2_ protocols and switch to top-fill methods and recommend a short delay following LOP before increasing CO_2_ flow rate. Furthermore, we highlight the benefit of utilizing neurophysiological methods alongside behavioral assessment to provide a robust, comprehensive welfare assessment to more reliably verify the conscious phase of concern. Collectively, our findings demonstrate that hypobaric hypoxia is a potential refinement to current methods for killing laboratory mice and may be potentially less harmful compared to hypercapnic hypoxia (that is, exposure to CO_2_). Our work demonstrates that gradual decompression could provide a practical, high-throughput alternative to CO_2_ and therefore could represent a major refinement for killing millions of laboratory mice worldwide. Future work should focus on determining the animals’ cognitive capabilities during the transition toward unconsciousness and the degree of active aversion in the conscious phase.

## Methods

### Ethics statement

All experiments were conducted at the University of Glasgow following ethical approval from the Animal Welfare and Ethical Review Body (AWERB) and project license approval from the Home Office (PPL:TP900S002; Protocol 2). Experiments were fully compliant with the EU Directive (2010/63/EU) and Animals Scientific Procedures Act (1986). All methods are reported in accordance with the ARRIVE guidelines for the reporting of animal experiments^95^.

### Animals, housing and husbandry

Twenty-four male C57BL/6J mice were obtained from Charles River UK with specific pathogen-free health status in accordance with the Federation of European Laboratory Animal Science Associations health monitoring recommendations. They were delivered at approximately 8 weeks of age across two batches (arrival dates 11 January 2022 and 8 February 2022). Mice were socially housed in pairs in filter top caging (1284L, Techniplast) with sawdust bedding (2HK Aspen wood chip, Datesand), nesting substrate (sizzle nest, Datesand) and a clear Perspex tunnel (Datesand Ltd) with food (BK001, Special Diet Services) and water available ad libitum. Mice were maintained on a 12:12 light–dark cycle (07:00 on, 19:00 off) under constant temperature (22 ± 1 °C) and humidity (48% ± 8%), in line with UK Home Office Code of practice guidelines^17^. All animals were acclimatized to the laboratory for at least 7 days before surgical implantation of subcutaneous EEG/ECG devices and commencement of experimental work. Mice weighed 24.5 g (minimum 21.3 g, maximum 28.1 g) at the time of surgery. All mice were handled using refined tunnel handling techniques to mitigate against background stress and anxiety^96^. All animals were checked daily, and their health was checked immediately before surgery.

### Induction of anesthesia and surgical preparation

After 1 week of acclimatization, mice underwent general anesthesia for surgical implantation of the subcutaneous wireless EEG/ECG transmitters (A3028B, Open Source Instruments). Mice underwent anesthetic induction via inhalation of vaporized 5% isoflurane (N01AB06, Piramal Critical Care) in 1 l/min oxygen in an induction chamber (World Precision Instruments). Once induced, the mouse was removed from the induction chamber and onto a standard rodent face mask and connected anesthetic rig (VetFlo Anaesthesia System), and the isoflurane was reduced to 1.5–2.5% in 1 l/min oxygen. The animal was then prepped for surgery, consisting of shaving the fur from the incision areas (top of the skull and one-quarter to three-quarters down the animals back). Preoperative analgesia (5 mg/kg carprofen, Rimadyl) and 0.10 mg/kg buprenorphine (Vetergesic) were administered subcutaneously into the flank of the animal^97^. The surgical sites were then disinfected before transfer to the surgical room.

### Surgical instrumentation of wireless transmitters

The animal was placed onto a stereotactic frame (Stoelting Europe) with heat pad and anesthetic face mask (1.5–2.5% isoflurane in 1 l/min oxygen) and was secured using a tooth bar. Once positioned, the mouse was then secured in the stereotactic frame using jaw cuffs (Stoelting Europe) to mitigate against potential ear trauma and tympanic membrane rupture^98^. Jaw cuffs were chosen as a refinement over the use of ear bars, fixing the head externally using the zygomatic plate rather than via the ear canals. Care was taken to consistently check sufficient anesthetic depth via pedal and corneal reflexes. Body temperature was monitored using a rectal temperature probe and oxygen saturation, respiration rate and heart rate were measured using a MouseOx (STARR Life Sciences). Once instrumented, the animal was draped, and lacri-lube (Allergan) was placed on each eye.

Surgery consisted of implanting a wireless battery powered dual-channel electrophysiology transmitter (A3028B, Open Source Instruments), which was disinfected with Reprodis HLD4i (Medimark Scientific Limited) before implantation. The main body of the device was implanted subcutaneously in the back of the animal, and the recording and reference leads were channeled under the skin to the skull. An incision was made to expose the skull and small craniotomies (0.9 mm) were drilled in two locations with intermittent irrigation using sterile saline: the reference electrode over the cerebellum (−6.00 mm caudal and 1.00 lateral to bregma) and the recording electrode over the motor cortex (M1/M2 bordering the somatosensory cortex S1HL, −1.00 mm caudal to bregma and 1.00 mm lateral to bregma). A small incision and subcutaneous pocket were made caudal to the shoulder blades and the body of the transmitter was placed into the subcutaneous pocket. The reference and recording leads were then channeled under the skin to the skull. The helical stainless-steel electrodes were placed in contact with the surface of the dura and secured into each craniotomy using miniature ground screws (Yahata, Neji, M1 pan head stainless-steel cross, RS Components). The second recording lead (for ECG) was secured to the left latissimus dorsi muscle using a small internal suture (Ethicon Sutures Vicryl 5/0). The skull implant and miniature screws were secured using dental cement (DuraLay Reliance), and the back incision was closed using sutures (Ethicon Sutures Vicryl 5/0).

### Postoperative care

Mice were allowed to recover from anesthesia in a heat cabinet (27–30 °C) in a recovery area and were closely monitored until they were fully conscious. After a minimum of 1 h recovery time where they were able to walk and move freely around the recovery cage, they were returned to their home cages. Postoperative buprenorphine was given in a palatable soft food (Nutella) immediately after surgery until 24 h after surgery (dose 0.10 mg/kg/day)^97,99^. All mice were habituated to the palatable soft food through daily presentation in their home cages for a minimum of 3 days before surgery^99^. Carprofen was also administered and available in the drinking water 24–48 h after surgery (dose: 20 mg/kg/day)^97,100^. All mice were monitored daily after surgery to ensure satisfactory wound healing, presence of normal behavior and maintenance of body weight. Mice were then allowed to recover for 8 days before exposure to one of the terminal treatments.

### Apparatus

A dual-purpose testing chamber was custom designed (Livetec Systems) and made from transparent acrylic (30 mm thick) with external dimensions including attachments of 610 mm (W) × 610 mm (D) × 470 mm (H) and internal dimensions of 400 mm (W) × 400 mm (D) × 400 mm (H). The chamber allowed application of both exposure to a gas (that is, CO_2_) and implementation of hypobaric hypoxia (gradual decompression), with a hose connected to a medical grade CO_2_ cylinder (201-D, BOC) and a central dual tap hose splitter to facilitate gas delivery or gradual decompression.

To achieve decompression, the chamber connects to an automated programmable logic controller system from which fully flexible programming of parameters are controlled to achieve a target decompression profile using a touch screen user interface. The chamber is connected to a vacuum pump (DVP lubricated rotary vane vacuum pump, LC25 2018) by hose and a proportional control valve enables fixed decompression rates, adjustable according to cycle time or pressure thresholds. Chamber pressure (mbar) was monitored and recorded every second using precision probes during baseline, decompression and recompression phases of the cycles. Following each cycle, recompression was facilitated manually over a period of at least 3 mins by an air admittance valve, monitored with a vacuum gauge. The pressure change during recompression was monitored and maintained by placement of a marker on the air admittance valve to ensure consistent recompression between individuals and was timed and recorded using a stopwatch. This ensured consistent, slow recompression at an overall average rate that was no faster than a descent equivalent to 65 ms^−1^.

For exposure to CO_2_, the chamber was connected to a second programmable logic controller, which was programmed to record the levels of CO_2_ and O_2_ gas every second. Both O_2_ and CO_2_ levels were monitored within the chamber at mouse height for each individual (Supplementary Fig. 2). The chamber was also fitted with multiple sensors to allow the continuous recording (every 1 s) of temperature (°C) and relative humidity (%). Given the gas inlet is at the top of the chamber, for CO_2_ bottom-fill, a custom attachment consisting of tubing and gas diffusion foam was attached to ensure delivery of the gas at two locations at the bottom of the chamber.

### Exposure to experimental terminal treatments

This study involved the exposure of mice to one of three experimental treatments: CO_2_ top-fill protocol, CO_2_ bottom-fill protocol and gradual decompression. The study followed a balanced, randomized-block factorial design using a Latin square. Blocking factors included batch and cage, to ensure mice housed in the same cage were killed in the same day. This design was fully balanced with respect to experimental treatment. Allocation of an individual mouse (nested within cage) were randomly allocated via a random number generator. Mice were exposed to their designated experimental treatment individually. Mice were removed from their home cage via refined handling techniques^96^ and were placed within the testing chamber, inside a custom-designed internal Perspex cube-shaped vivarium 20 cm (W) × 20 cm (D) × 20 cm (H) (inner dimensions; Livetec Systems), with the floor lined with clean Vetbed. All four sides (clear Perspex, ~0.3 mm thickness) of the vivarium were perforated with two rows of ~0.5 mm circular holes, at ~2-cm intervals, at approximately mouse height and the lowest row flush with the Vetbed lining. The lid of the vivarium (black Perspex, ~0.3 mm thickness), was fully perforated with a total of twenty-five ~0.5 mm circular holes, stratified into a grid pattern. As part of the design and development of the testing chamber and internal vivarium for consistent application of all three treatments to the desired protocols, gas sensors (O_2_ and CO_2_) were used to monitor gas concentrations throughout both areas and to ensure the ambient atmosphere at mouse height within the internal vivarium matched the equivalent outside the internal vivarium (but within the testing chamber).

Following treatment application and animal removal, the used Vetbed within the vivarium was removed, and the internal surfaces cleaned, disinfected and thoroughly dried, before replacement of a fresh Vetbed lining (that is, clean and laundered). This was undertaken for each individual, to mitigate against potential crossover of odor cues^101^. Following a 30-s baseline period, animals were exposed to one experimental treatment.

### Gradual decompression

In line with previous work^44,45^, the applied decompression profile consisted of two distinct phases. Phase 1 involved decompression from sea level (1,013 mbar) to a target pressure of 200 mbar (~11,800 m equivalent altitude) followed by a subsequent hold phase (phase 2) for a minimum of 5 min and confirmation of death (that is, >30 s motionless). The rate of decompression was relatively consistent, with the 200-mbar pressure target (phase 1) reached in 294 s (288–298 s), resulting in an average 40.1 ms^−1^ (39.6–41.0 ms^−1^) equivalent rate of ascension to 11,800 m. Decompression rate was based on previous findings^44,45^ whereby a linear and slow rate of decompression was employed (~40 ms^−1^). The mean total cycle time (phases 1 and 2) was 594 s (range 588–598 s).

### Carbon dioxide (CO_2_)

In line with current best practice guidelines in the UK and Europe^32,34^, CO_2_ was applied via a medical grade 100% CO_2_ cylinder (201-D, BOC), at a flow rate of approximately 20% of the chamber volume per minute, which was introduced to the top of the chamber, mediated manually via a flowmeter. Gas flow was maintained at 20% volume per minute until LOP (CO_2_ top-fill mean: 116 s, range 98–130 s, CO_2_ bottom-fill mean: 115 s, range 104–143 s), at which point it was then increased to 80% of the chamber volume per minute until observed cessation of breathing (CO_2_ top-fill mean: 231 s, range: 211–256 s, CO_2_ bottom-fill mean: 192 s, range: 171–222 s). Upon respiratory arrest, a subsequent 2-min dwell time ensued resulting in a mean total cycle time of (CO_2_ top-fill mean: 351 s, range: 331–376 s, CO_2_ bottom-fill mean: 312 s, range: 291–342 s).

### Assessment of the tympanic membrane

Following CO_2_ and gradual decompression terminal treatments only and upon removal from the chamber, death was secondarily confirmed in accordance with Schedule 1 through permanent cessation of the circulation by severing the femoral artery for all mice. Tympanic membranes were assessed by examination of the external ear canals with a stereomicroscope, and the tympanic membranes were recorded as intact or ruptured for all mice undergoing CO_2_ and gradual decompression terminal treatments (*n* = 8 per terminal treatment).

### Behavioral observations

The behavior of each mouse was video recorded using a GeoVision surveillance system (GV800B) connected to four wired cameras (Bird box camera 1080p with IR Night vision) sitting at different positions outside of the decompression chamber, providing a full bilateral cranial–caudal view. The system allowed direct live monitoring of the mouse on an external monitor, enabling live animal welfare assessment and informed actions of humane endpoints if required. In addition, all captured video footage was stored and backed up for future in-depth analysis. Behavioral footage for each mouse was retrospectively observed and analyzed using Noldus Observer XT (version 12) by a single blinded observer. A comprehensive ethogram (Supplementary Table 1) was developed in line with previous work on gaseous killing methods in laboratory mice^44,82,87,102,103^. We took a mechanistic approach to ethogram design to avoid presumptions of motivational drive and minimize observer interpretation of intention. Behavioral variables measured included latencies, counts and total durations. Data were exported from Noldus Observer to Microsoft Excel.

### Statistical analyses

Statistical analyses were conducted in R (v 4.0.3, R Core Team) via the RStudio platform (Version 1.3.1093, RStudio, PBC, 2009–2020). All data were collated and processed within R using the tidyverse package^104^. All graphical summaries were created using the ggplot2 package^105^. Behavioral data (latencies, counts, durations and so on) and processed iEEG and ECG data (latencies, spectral characteristics and so on) were analyzed with generalized linear mixed models (GLMMs) via the glmmTMB package^106^ and general linear mixed models via the lme4 package^107^ and were used to identify fixed effects that may affect each measure. Model fit was determined by examination of residuals via the DHARMa package^108^ and appropriate error distributions set for GLMMs. Binary variables (for example, behavioral occurrence (0/1) per individual) were analyzed with the family link set to binomial. Subset analyses were performed on infrequent behaviors including counts for ear scratches, head flicks, head shakes and escape attempts to determine the relationship of fixed effects on the likelihood of occurrence, only across animals that performed them. All models included fixed effects of treatment (three levels: decompression, CO_2_ bottom-fill or CO_2_ top-fill). Interactions between fixed effects were included in the model. Relative humidity and temperature were included in the model as covariates to ensure they did not have a significant effect upon outcome variables and removed if non-significant to improve model fit. All models included the covariate mouse weight (accounting for weight differences) and random nested effects, with unique cage number nested within batch thereby controlling for non-independence of mice housed in the same cage and for potential variation between batches (for example, animal source or litter effects). Statistical significance based on the *P* < 0.05 threshold was calculated using the ANOVA function (car package^109^) to ascertain differences derived from fixed effects and interactions, and only statistically significant results are reported. Pairwise comparisons were reported using estimated marginal means via the emmeans package^110^, with *P* values adjusted for multiple comparisons using the Tukey method.

### Data processing and analysis

#### iEEG data

Wireless iEEG data were recorded using LWDAQ software (Open Source Instruments) with a sampling rate of 512 Hz. Data were exported from LWDAQ software and imported into Labchart V8 (ADInstruments).

Spectral analyses consisted of manually examining 1-s epochs from the iEEG signals alongside behavioral assessments using video footage to identify and eliminate movement artifacts. Power spectra analysis was performed using a fast Fourier transform (1024, Hanning window, frequency resolution of 0.5 Hz). Two spectral variables were then calculated in Labchart V8, PTOT and F50. PTOT is defined as the total area under the power spectrum curve, thus representing the total power of all frequencies^8,9,11,94^. F50 represents the frequency below which 50% of the total power of the EEG is located^8,9,11,94^ and, as such, provides a quantitative means of determining the dominant frequency band.

Time–frequency decomposition analysis was performed using the Fieldtrip toolbox (Oostenveld, 2011; http://fieldtriptoolbox.org) in MATLAB. Data were preprocessed using the Fieldtrip toolbox (Oostenveld, 2011; http://fieldtriptoolbox.org) in MATLAB and were demeaned, detrended and low-pass filtered (30 Hz). Artifact removal was achieved using a two-step process. First, a variational mode decomposition method was employed in MATLAB, with the intrinsic mode function (IMF) parameters tuned to best denoise the signal (IMFs of 22, final two modes removed)^111,112^. Second, large, nonbiological noise was removed by *z*-transforming the signal, identifying time points breaching a threshold (*Z* > 6), cutting a 50-ms window around the artifact, and locally interpolating across the missing signal. Fourier analysis was performed on each trial of preprocessed data (Hanning window, 4-s sliding window, frequency range of 1–13 Hz, no baseline correction) to produce time–frequency representations of power. Trials were then averaged in the time–frequency domain to preserve induced responses (non-time and phase locked components), and the average time–frequency responses were normalized across conditions.

#### ECG data

ECG data were recorded using LWDAQ software (Open Source Instruments) with a sampling rate of 512 Hz. Data were exported from LWDAQ software and imported into MATLAB. A variational mode decomposition method was employed in MATLAB, with the IMF parameters tuned to best denoise the signal (IMFs of 9, retaining modes 2–4 inclusive)^113^. Data were then imported into Labchart V8 (ADInstruments). Heart rate was automatically detected using the HRV module (2.3) (ADInstruments). All waveforms and heart rate peaks were then manually checked, and misclassified beats were manually removed. Heart rate was sampled every second throughout the duration of each terminal treatment, including a 30-s baseline period before cycle onset.

### Reporting summary

Further information on research design is available in the Nature Portfolio Reporting Summary linked to this article.

## Online content

Any methods, additional references, Nature Portfolio reporting summaries, source data, extended data, supplementary information, acknowledgements, peer review information; details of author contributions and competing interests; and statements of data and code availability are available at https://doi.org/10.1038/s41684-026-01788-5.

## Supplementary information

Supplementary Information Supplementary Figs. 1 and 2 and Table 1.

Reporting Summary

## Data Availability

Data supporting this study are available via Zenodo at https://doi.org/10.5281/zenodo.21220901 (ref. ^114^).
