# Supplementary material for: Neural and behavioral correlates of consciousness: the welfare consequences of fatal hypobaric and hypercapnic hypoxia in laboratory mice
Source: Lab Anim (NY). 2026 Aug 21;55(11):429–40. doi: 10.1038/s41684-026-01788-5 (PMC13710156; doi:10.1038/s41684-026-01788-5)
Supplement: Supplementary file 1 [file 41684_2026_1788_MOESM1_ESM.pdf]

lab animal

https://doi.org/10.1038/s41684-026-01788-5

Neural and behavioral correlates of consciousness: the welfare consequences of fatal hypobaric and hypercapnic hypoxia in laboratory mice

In the format provided by the authors and unedited

Supplementary Information for "Neural and behavioural correlates of consciousness: the welfare consequences of fatal hypobaric and hypercapnic hypoxia in laboratory mice"

Jasmine M. Clarkson[1,2*], Dorothy E.F. McKeegan[1], Ross S. Muers[3,4], Matthew C. Leach[5], Julian. Sparrey[6], Alfredo. Gonzalez-Sulser[7] and Jessica E. Martin[2,8]

[1] School of Biodiversity, One Health and Veterinary Medicine, College of Medical Veterinary and Life Sciences, University of Glasgow, Glasgow, UK

[2] School for Natural and Environmental Sciences, Newcastle University, Newcastle Upon Tyne, UK

[3] Centre for Neuroscience, School of Psychology and Neuroscience, College of Medical Veterinary and Life Sciences, University of Glasgow, Glasgow, UK

[4] SRUC Dairy Research and Innovation Centre, Barony Campus, Dumfries, UK

[5] Comparative Biology Centre, Newcastle University, Newcastle Upon Tyne, UK

[6] Livetec systems Ltd, Wrest Park, Silsoe, Bedfordshire, UK

[7]Simons Initiative for the Developing Brain, Patrick Wild Centre and Centre for Discovery Brain Sciences, University of Edinburgh, Edinburgh, UK

[8] The Royal (Dick) School of Veterinary Studies and The Roslin Institute, The University of Edinburgh, Edinburgh, UK

Corresponding author*: jasmine.clarkson@newcastle.ac.uk

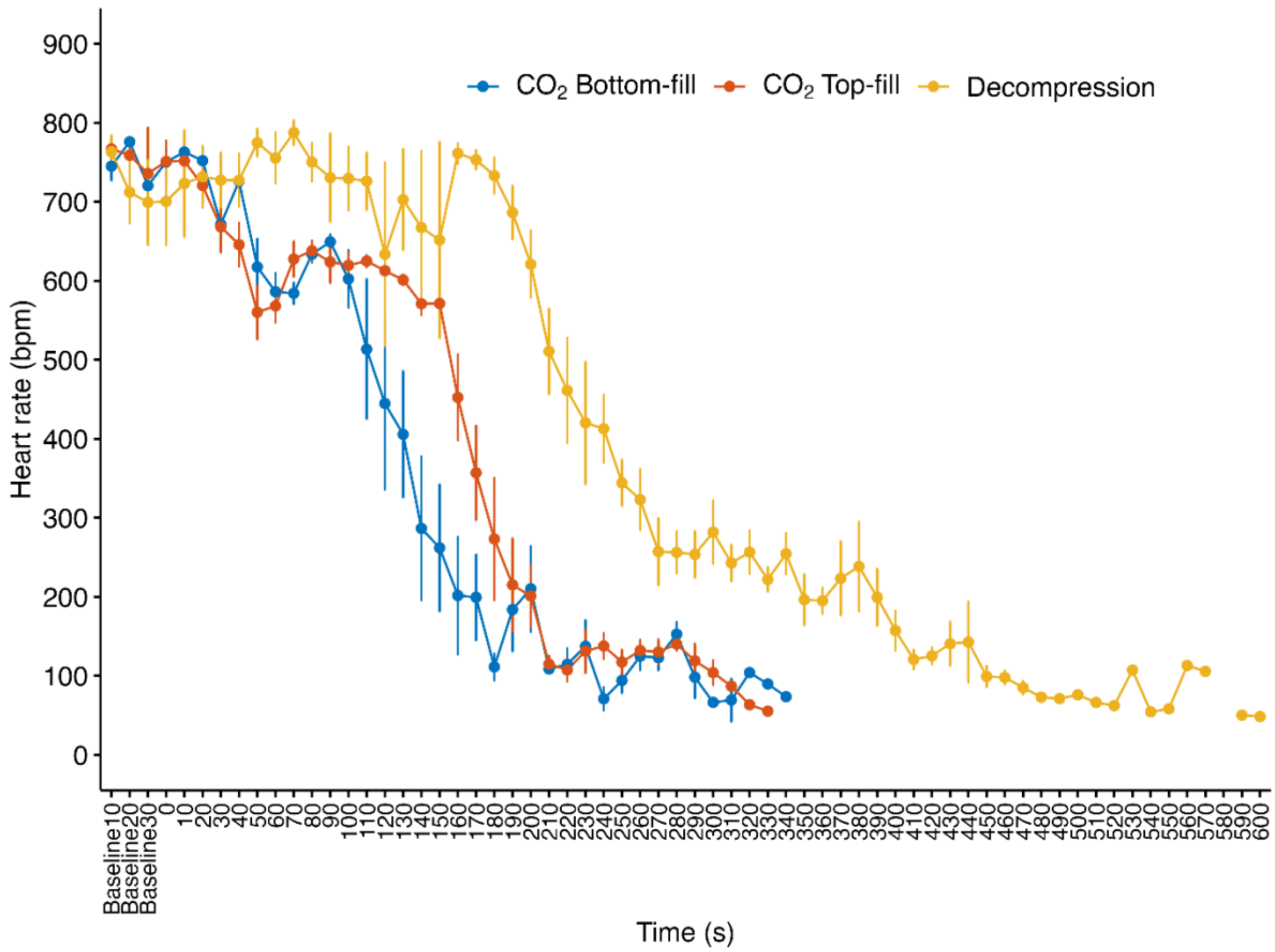

Figure S1: Effect of terminal treatments on heart rate

Mean ± SE heart rate (bpm) obtained from ECG traces according to terminal treatment, blue denotes bottom filled CO_2, red denotes top filled CO_2, yellow denotes gradual decompression.

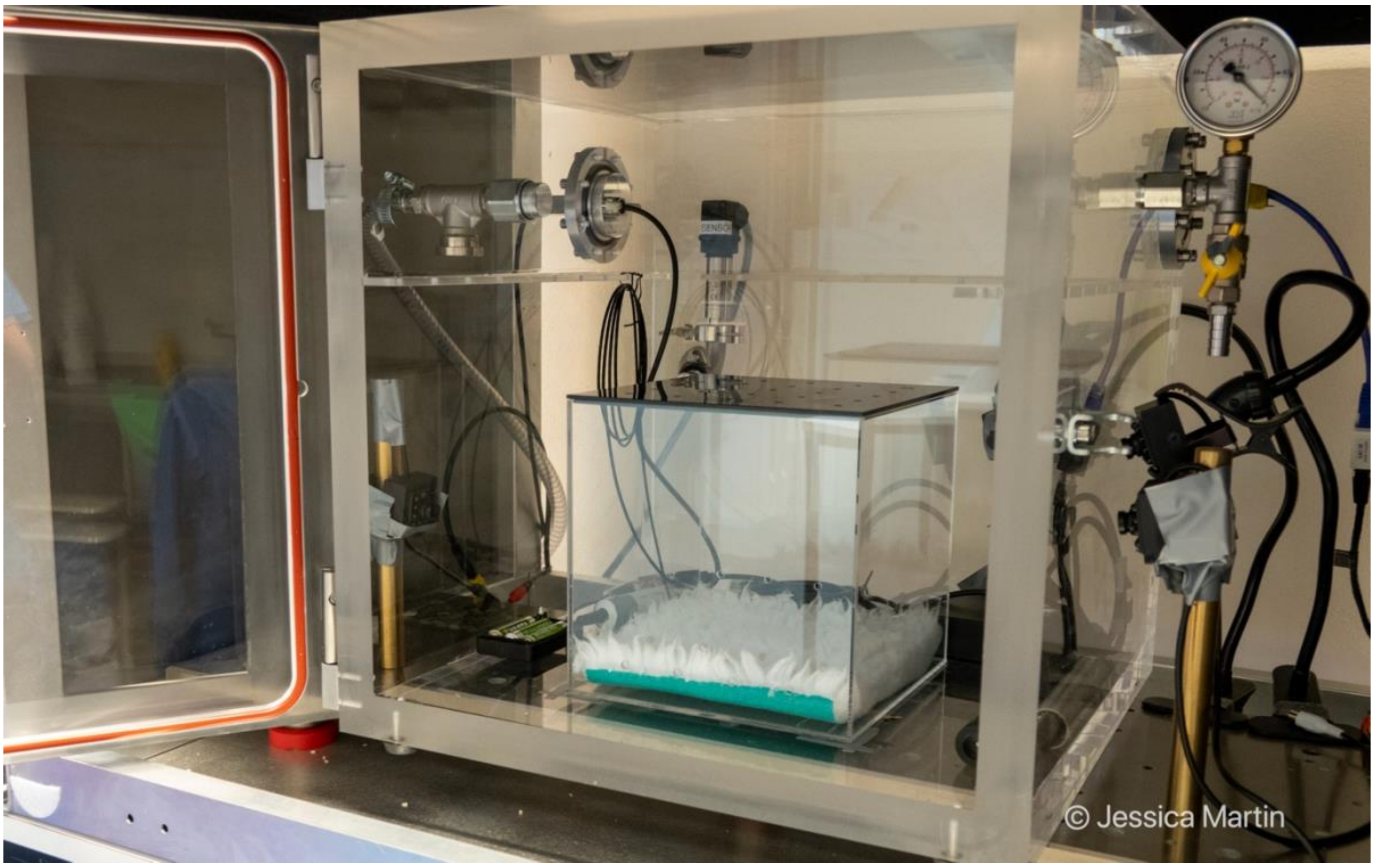

Figure S2: Photograph of experimental setup

Chamber consisting of outer acrylic chamber and inner Perspex vivarium. Multiple wide-angle cameras located on three sides of the chamber allowed for full bilateral cranial–caudal view of the mouse and facilitated complete behavioural observations. Additional sensors including pressure, temperature, relative humidity and carbon dioxide sensors, as well as a microphone for ultrasonic vocalization analysis were also present.

Table S1: Summary of Behavioural ethogram

Behavioural ethogram outlining key behaviours scored during analysis in Observer XT (Noldus, version 12).

Category	Description (including modifier(s))	Behaviour Type
**RESPIRATORY MARKERS**	**Assessment of the respiratory condition and mouth orientated behaviours**	**Mutually exclusive, exhaustive**
Normal rhythmic breathing	Regular rhythmic movement of the thoracic/diaphragm area, usually with closed mouth	State – initial state event
Abnormal breathing	Deviation from normal rhythmic breathing and can be defined as:<br>Fast – Increased respiratory rate from normal rhythmic breathing<br><br>Slow – Decreased respiratory rate from normal rhythmic breathing<br><br>Irregular – non-rhythmic respiration with erratic movement of the thoracic/diaphragm area causing irregular movement. Mouth may be open or opened and closed at intervals.	State
Gasping	Oral breathing. Mouse opens mouth and holds mouth open to breath, opening and closing at intervals of differing times, fast or slow. Often accompanied by larger movement of the thoracic/diaphragm area.	Point event
Apnoea	Cessation of breathing -No regular/rhythmic movement of the thoracic/diaphragm area, or the mouth, spasmodic movements may occur indicative of agonal gasps caused by brainstem reflex/myoclonus.	State
Agonal gasp	Post apnoea, mouse involuntarily opens and closes the mouth, in a distinct abnormal pattern, usually whilst recumbent. Often includes other body reflexes including the head raising and obvious movement of the thoracic/diaphragm area.	Point event
Last agonal gasp	Last time an agonal gasp movement occurred	Point event
**POSTURES**	**Assessment of the physical stance and function**	**Mutually exclusive, exhaustive**
Stand	Supported upright by all four legs and is stood still or shifting weight from foot to foot but without travelling.	State – initial state event
Lie	Lowers body to the floor so that the belly, side and/or sternum are in contact with the floor. Legs are either bent or straight. Ventral or lateral (out with another behaviour i.e LOP)	State
Freeze	Complete cessation of movement, alert with ears in upright position for >3s and occurring before LOP.	State
Stretched attend posture	Investigatory posture – mouse braces and pushes forward with its hind legs, stretches its body forward with the belly pressed against the ground with head raised.	State & Point event
Early ataxic	Onset of incoordination of body movements, head/body swaying, drowsy, weight distribution through tip toes.	State
Loss of balance (LOB)	Loss of control of voluntary actions and stability, unable to remain on all 4 feet, stumbling with the inability to rectify position to some degree. Postural changes may occur as disorientation occurs.	State

Loss of posture (LOP)	Inability to retain posture even through active endeavour. All legs are unable to support weight. May be accompanied by other behaviours (i.e. leg paddling, head movements)	State
Convulsion	Sudden, forceful, irregular movement of the body, caused by involuntary rapid contraction and relaxation of body muscles repeatedly (clonic), resulting in an uncontrolled shaking, jerking and flipping of the body, loss of limb function and head control.	State
Convulsion intensity	Measure of magnitude and intensity of the convulsion period itself whereby, in order of severity:<br><br>Low-medium severity includes:<br>Flick twitch – singular, slight, small movement<br>Spasm – Occasionally initially but generally a second or third convulsion bout where the body shudders but remains on floor lateral or ventral<br>Rise up – In clonic state body reflexes cause the mouse to sit or draw up from the abdomen as if into a sitting posture but legs/head are limp and posture is unsustainable when muscles release<br><br>High severity includes:<br>Lateral thrash – Side to side head/body/leg movements in very rapid succession whilst in lateral position where body remains floor bound<br>Lateral 360 – Body is rotated in a circular motion in lateral posture between 180-360°<br>Back flip – rotation of body backwards, head over spine in vertical trajectory<br>Airborne – whole body leaves the floor<br>Ejection – Body/head hits the top of the chamber	Point event
Recumbent	Prone/prostrate, lateral, ventral or dorsal, having lost full control of postural function and in semi or unconscious state lying down or propped up by box.	State
Motionless	Cessation of all movement. Modifier for the body position of final rest.	State
**MOUSE BEHAVIOUR**	**Assessment of the behaviour and physiological function of the mouse**	**Start-Stop (behaviours can overlap)**
Exploration	General exploratory locomotion, mouse moving around the chamber.	State
Rear	The mouse puts its weight on its hind legs, raises its forelimbs from the ground, and extend its head upwards.	State & point event
Inactive	Natural relaxed posture (face lifted whilst curled up) showing little movement, not engaged in exploration of environment, may look fatigued, drowsy, uninterested, or calm but appears responsive to external sounds.	State
Escape attempt (jump)	Upright motion towards the chamber lid where all four paws are off the floor occurring before LOP. This can occur during rearing where the mouse performs a small jump or can be independent of rearing.	Point event
Ear scratch	Mouse scratches ear with hind paw.	State
Tail lift	Tail is lifted from the normal stance, lifted off the floor in a vertical direction.	State & Point event
Circling	A circling mouse repetitively traces a loosely circular path. While circling, the mouse may change its body	State

	orientation, alter its direction, and intermix other behaviours while following the path.	
Undirected sniffing	Investigatory behaviour with the nose held in the air and away from any identifiable stimulus.	State
Grooming bout	Usually in a sitting position, the mouse will lick its fur, groom with the forepaws or scratch with any limb. Typical grooming consists of a mix/sequence of the four behaviours outlined in 'grooming component'. This behaviour consists of multiple of these behaviours rather than a single component alone.	State
Grooming component	Usually in a sitting position, the mouse will lick its fur, groom with the forepaws or scratch with any limb. Typical grooming consists of a mix/sequence of the four behaviours:<br><br>Abnormal grooming consists of short, interrupted grooming that does not consist of a mix/sequence of these four behaviours.<br>Elliptical Stroke – Elliptical asymmetric movements of the forepaws over the nose and muzzle, alternating the major and minor paw.<br>Unilateral Stroke – Alternating strokes of the forepaw across the vibrissae and the eye.<br>Bilateral Stroke – Large symmetric bilateral strokes of the forepaws that begin behind the ears and pass over the whole face.<br>Body Licking – Licking of the whole body, typically beginning rostrally and working caudally to the tail.	Point event
Facial grimace	Pre-recumbency changes to one or more facial attributes (Facial action units) of the mouse grimace scale with classified severity (moderately present or obviously present).<br><br>Orbital tightening – closing of the eyelid (narrowing of orbital area)<br>Nose bulge – bulging of the bridge of the nose with vertical wrinkles on the side of the nose<br>Cheek bulge – bulging of the cheeks<br>Ear position – Ears rotate outwards and/or backwards, away from the face. May fold in a pointed shape. Position between the ears increases.<br>Whisker change – whiskers are either pulled back against the cheek or pulled forward to 'stand on end'. May clump together. Lose their natural downward curve.	State & Point event
Head shake	Quick, sudden rotational movements left and right (side to side) isolated to the head.	Point event
Head tilt	Head is held to one side or the other or swapped from side to side (slower than a head shake)	State & Point event
Head flick	Quick, sudden movement of the head in a rapid forceful action.	Point event
Head up	Head is held high with nose at the highest point.	State & Point event
Head lift	During recumbency, head is lifted off the floor or moved/repositioned by use of the neck not through spasm.	Point event
Dig	A series of fast alternating movements of the forepaws scraping at the vet-bed.	State
Urinate	Passing of urine either voluntarily or involuntary (loss of bladder control) post recumbency.	Point event

Defecate	Passing of faeces	Point event
Abdominal swelling	Abdominal area begins to swell.	Point event

NB: Mutually exclusive denotes behaviours cannot happen at the same time. Exhaustive refers to behaviour being fully comprehensive, point event refers to a single, and often brief behavioural event, state events refer to behaviours where behaviours are longer lasting and durations can be calculated.
