# Supplementary material for: Neural and behavioral correlates of consciousness: the welfare consequences of fatal hypobaric and hypercapnic hypoxia in laboratory mice
Source: Lab Anim (NY). 2026 Aug 21;55(11):429–40. doi: 10.1038/s41684-026-01788-5 (PMC13710156; doi:10.1038/s41684-026-01788-5)
Supplement: Supplementary file 2 [file 41684_2026_1788_MOESM2_ESM.pdf]

nature portfolio

Corresponding author(s): Dr Jasmine M Clarkson

Last updated by author(s): Jul 7, 2026

Reporting Summary

Nature Portfolio wishes to improve the reproducibility of the work that we publish. This form provides structure for consistency and transparency in reporting. For further information on Nature Portfolio policies, see our Editorial Policies and the Editorial Policy Checklist.

Statistics

For all statistical analyses, confirm that the following items are present in the figure legend, table legend, main text, or Methods section.

n/a	Confirmed	
☐	☒	The exact sample size (*n*) for each experimental group/condition, given as a discrete number and unit of measurement
☐	☒	A statement on whether measurements were taken from distinct samples or whether the same sample was measured repeatedly
☐	☒	The statistical test(s) used AND whether they are one- or two-sided<br>*Only common tests should be described solely by name; describe more complex techniques in the Methods section.*
☐	☒	A description of all covariates tested
☐	☒	A description of any assumptions or corrections, such as tests of normality and adjustment for multiple comparisons
☐	☒	A full description of the statistical parameters including central tendency (e.g. means) or other basic estimates (e.g. regression coefficient) AND variation (e.g. standard deviation) or associated estimates of uncertainty (e.g. confidence intervals)
☐	☒	For null hypothesis testing, the test statistic (e.g. F, t, r) with confidence intervals, effect sizes, degrees of freedom and P value noted<br>*Give P values as exact values whenever suitable.*
☐	☒	For Bayesian analysis, information on the choice of priors and Markov chain Monte Carlo settings
☐	☒	For hierarchical and complex designs, identification of the appropriate level for tests and full reporting of outcomes
☐	☒	Estimates of effect sizes (e.g. Cohen's d, Pearson's r), indicating how they were calculated

Our web collection on statistics for biologists contains articles on many of the points above.

Software and code

Policy information about availability of computer code

Data collection	Wireless iEEG and ECG data was recorded using LWDAQ software (Open Source Instruments, USA) with a sampling rate of 512Hz. Behavioural data was captured using GeoVision surveillance system (GV800B) connected to four wired cameras (Bird box camera 1080p with IR Night vision).
Data analysis	iEEG and ECG data was exported from LWDAQ software and imported into Labchart V8 (ADInstruments, Australia). Time-frequency decomposition analysis for iEEG data was performed using the Fieldtrip toolbox (Oostenveld, 2011; http://fieldtriptoolbox.org) in MATLAB. Statistical analyses were conducted in R (v 4.0.3, R Core Team) via the R Studio platform (Version 1.3.1093, RStudio, PBC, 2009-2020). Behavioural data was analysed using continuous sampling methods using Noldus Observer XT (version 12) by a single blinded observer.

For manuscripts utilizing custom algorithms or software that are central to the research but not yet described in published literature, software must be made available to editors and reviewers. We strongly encourage code deposition in a community repository (e.g. GitHub). See the Nature Portfolio guidelines for submitting code & software for further information.

Data

Policy information about availability of data

All manuscripts must include a data availability statement. This statement should provide the following information, where applicable:

- Accession codes, unique identifiers, or web links for publicly available datasets
- A description of any restrictions on data availability
- For clinical datasets or third party data, please ensure that the statement adheres to our policy

All data generated in and analysed during the current study will be publicly available in Zenodo repository (DOI will be made available upon article revision/ acceptance).

Field-specific reporting

Please select the one below that is the best fit for your research. If you are not sure, read the appropriate sections before making your selection.

- [x] Life sciences
- [ ] Behavioural & social sciences
- [ ] Ecological, evolutionary & environmental sciences

For a reference copy of the document with all sections, see nature.com/documents/nr-reporting-summary-flat.pdf

Life sciences study design

All studies must disclose on these points even when the disclosure is negative.

Sample size	Sample size was determined A priori using data from previously published work (Clarkson et al. (2023.) 'Striving for Humane Deaths for Laboratory Mice: Hypobaric Hypoxia Provides a Potential Alternative to Carbon Dioxide Exposure'. Proceedings of the Royal Society B 290 (1997). https://doi.org/10.1098/RSPB.2022.2446). Sample size analyses were conducted in R using the general linear model (pwr package). To detect the smallest estimated effect sizes observed in previous data, with a desired power set at 0.8, significance level 0.05, and three coefficients included in the model, a sample size of n=7 mice per treatment were estimated.
Data exclusions	iEEG data from two mice (one decompression and one CO2 bottom) were excluded due to floating electrode artefact.
Replication	To facilitate reproducibility of findings, all methods outlined in the paper are described in accordance with the ARRIVE guidelines. In addition, all primary and secondary data and its respective code associated with data analysis will be made freely available upon publication of the article in a suitable repository.
Randomization	Mice were randomised to treatment allocation according to a balanced randomised-block factorial design using a Latin square. Blocking factors included batch and cage to ensure mice housed in the same cage were killed on the same day. This design was fully balanced with respect to experimental treatment.
Blinding	Investigators could not be blinded to treatment group during data collection, however, for data analysis all data files were coded to ensure blinding with regards to treatment.

Reporting for specific materials, systems and methods

We require information from authors about some types of materials, experimental systems and methods used in many studies. Here, indicate whether each material, system or method listed is relevant to your study. If you are not sure if a list item applies to your research, read the appropriate section before selecting a response.

Materials & experimental systems

n/a	Involved in the study	
☒	☐	Antibodies
☒	☐	Eukaryotic cell lines
☒	☐	Palaeontology and archaeology
☐	☒	Animals and other organisms
☒	☐	Human research participants
☒	☐	Clinical data
☒	☐	Dual use research of concern

Methods

n/a	Involved in the study	
☒	☐	ChIP-seq
☒	☐	Flow cytometry
☒	☐	MRI-based neuroimaging

Animals and other organisms

Policy information about studies involving animals; ARRIVE guidelines recommended for reporting animal research

Laboratory animals	Twenty-four male C57BL/6J mice were obtained from Charles River UK at approximately eight weeks of age.
Wild animals	The study did not involve wild animals.
Field-collected samples	The study did not involve samples collected from the wild.
Ethics oversight	All experiments were conducted at the University of Glasgow following ethical approval from the Animal Welfare and Ethical Review Body (AWERB) and project license approval was granted from the UK Home Office (PPL:TP900S002; Protocol 2). Experiments were fully compliant with the EU Directive (2010/63/EU) and Animals Scientific Procedures Act (1986), UK. All methods are reported in accordance with the ARRIVE guidelines for the reporting of animal experiments.

Note that full information on the approval of the study protocol must also be provided in the manuscript.
